# Acetaminophen influences social and economic trust

**DOI:** 10.1038/s41598-019-40093-9

**Published:** 2019-03-11

**Authors:** Ian D. Roberts, Ian Krajbich, Baldwin M. Way

**Affiliations:** 1 Department of Psychology, University of Toronto, 1265 Military Trail, Toronto, Ontario M1C 1A4 Canada; 2 Department of Psychology, The Ohio State University, 1835 Neil Avenue, Columbus, OH 43210 USA; 3 Department of Economics, The Ohio State University, 1945 N High Street, Columbus, OH 43210 USA; 4 Institute for Behavioral Medicine Research, The Ohio State University, 460 Medical Center Drive, Columbus, OH 43210 USA

## Abstract

Acetaminophen has long been assumed to selectively alleviate physical pain, but recent research has started to reveal its broader psychological effects. Building on this work, we find suggestive evidence that acetaminophen affects the basic social process of trust across a national survey and five lab experiments. In a national community sample (MIDUS II), acetaminophen usage was negatively associated with neighborhood trust and feelings of social integration. In a series of lab experiments (N = 767), acetaminophen reduced the influence of self-generated expectations on investments in a trust game. When we manipulated trust game investor expectations, acetaminophen increased investments regardless of expectations. These results provide the first demonstration that an over-the-counter drug can impact trust-related behavior. Overall, the findings paint a complex picture of how situational factors may influence drug effects.

## Introduction

Acetaminophen (i.e., paracetamol; the active ingredient in Tylenol) has long been known to be an analgesic and antipyretic. However, a growing body of work is finding that the effects of acetaminophen extend beyond physical pain and fevers to psychological processes. It is therefore natural to ask whether acetaminophen might affect people’s social behavior. This is important because acetaminophen is the most commonly used pain medication in the United States with 23% of the population taking it each week^1^. If acetaminophen impacts social behavior, there could be broad implications from the personal to the societal level.

Trust is commonly studied in behavioral economics because it is a critical component of social relationships^2^, and is vital for the functioning of the institutions that form the backbone of large-scale integrated societies such as governments, businesses, and religions^3,4^. Acetaminophen is of interest because it has recently been found to influence social and affective processes, on which these behaviors are thought to rely. For example, acetaminophen reduced self-reported hurt feelings and neural responses associated with experiences of social pain^5,6^. It also reduced distress about another person’s physical and social misfortunes^7^. Recently, acetaminophen was shown to reduce affective responses to both negative and positive emotional images^8^. Based on these findings, we sought to examine whether acetaminophen would have effects on trust.

Here we investigate the effects of acetaminophen on trusting behavior using a combination of survey data on interpersonal trust and social integration, and laboratory experiments using a social preference game from experimental economics, the trust game. We find that acetaminophen use is associated with reduced neighborhood trust and feelings of social integration. In a series of laboratory experiments, we find that an acute dose of acetaminophen reduces the correlation between expectations about trustee repayment and investments in the trust game. Based on this result of acetaminophen reducing the correspondence between expectations and behavior, we ran larger experiments with a trust game in which we manipulated expectations about the trustees. In this context, we found that acetaminophen increased behavioral trust, regardless of expectations. Our results point to possible effects of acetaminophen on trust-related behavior and underscore the likely important influence of various contextual and environmental factors on the effects of this drug.

## Results

### Survey Data

To test whether there might be a relationship between regular acetaminophen usage and interpersonal trust, we examined a national survey dataset (MIDUS II) where a subset of participants (N = 3,410) reported on their frequency of acetaminophen usage in the past 30 days and completed a 4-item scale assessing their perceptions of neighborhood trust and safety^9^. The items were “People in my neighborhood trust each other”, “I could call on a neighbor for help if I needed it”, “I feel safe being out alone in my neighborhood during the daytime”, and “I feel safe being out alone in my neighborhood at night.” Additionally, if acetaminophen has effects on a fundamental social process such as trust, then it could influence the quality of interpersonal relationships that people have with others. Therefore, we also examined the relationship between acetaminophen use and a 3-item measure of feelings of social integration with their community^9^. The items were “I don’t feel I belong to anything I’d call a community”, “I feel close to other people in my community”, and “My community is a source of comfort”.

**Figure 1:**
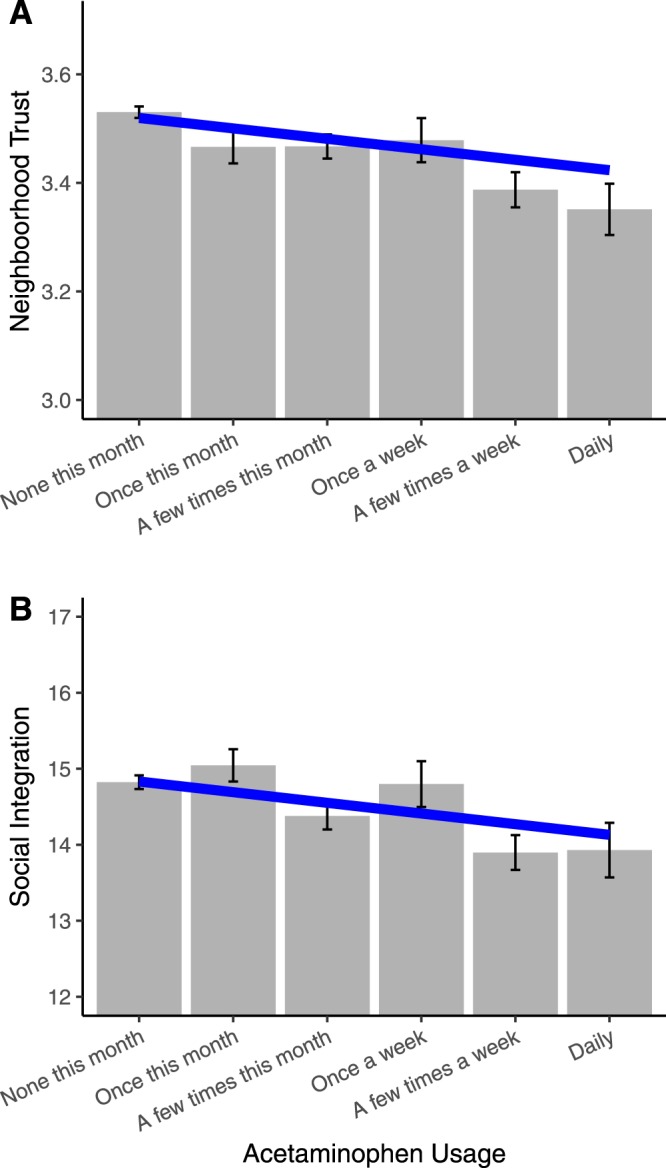
Survey data results for acetaminophen usage and (**A**) neighborhood trust and (**B**) social integration. Error bars represent standard error of the mean.

We controlled for several covariates in our analyses: sex, age, education, and overall household income. Because many individuals take acetaminophen to relieve pain, we also included a dichotomous measure assessing the presence of chronic pain as a covariate. Even when controlling for these covariates, there was a significantly negative relationship between acetaminophen usage and perceived neighborhood trust (*b* = −0.06 (0.02), *t* = −3.46, *p* < 0.001, BCA 95% CI: [−0.10, −0.02]; see Fig. 1A). There was also a significantly negative relationship between acetaminophen usage and social integration (*b* = −0.06 (0.02), *t* = −3.07, *p* < 0.001, BCA 95% CI: [−0.09, −0.02]; see Fig. 1B). In additional analyses, we controlled for other covariates (see Tables S2 and S3).

While these results are suggestive, they do not provide evidence of causality. It could be that low-levels of neighborhood trust and social integration lead people to self-medicate with pain-killers^5,6^. Alternatively, a third variable such as depression or stress could cause both reduced social integration and increased acetaminophen usage. Therefore, laboratory experiments are necessary to test whether acetaminophen use itself might influence trust behavior.

### Laboratory Experiments

To test whether acetaminophen might influence trust, we conducted a series of lab experiments to investigate the effects of an acute dose of 1000 mg of acetaminophen (the standard extra strength dose for a headache) on trust game investors as part of a battery of social economic decision-making tasks (see Supplemental Material available for results from dictator game, ultimatum game as proposer and responder, and trust game as trustee). In all these experiments, participants were informed that one of their decisions would be selected at random and both they and their anonymous partner would be sent money based on their choices.

#### Trust game as investor; No information on partner’s past behavior

In Experiments 1, 2, and 3, participants completed a widely-used, economic measure of trust—investor behavior in a trust game (TG-I)^10^. The TG-Is used in Experiments 1, 2, and 3 were highly similar (see the Methods for a full description of differences). On each trial, participants were given a monetary endowment and then permitted to transfer as much or as little as they liked to an anonymous partner (i.e., the trustee). Any amount that was transferred was multiplied by four before being given to the trustee. While past work has demonstrated that one important factor that contributes to an individual’s level of trust is aversion to the possibility of betrayal^11^, expectations about trustee reciprocity also have a significant influence on trust behavior^12^. Therefore, on each trial, immediately after deciding how much to transfer to the trustee, participants reported how much they expected the trustee to return (i.e., expected return). In Experiment 1, participants advanced to the next trial after reporting their expectations, but in Experiments 2 and 3 they also evaluated their anticipated affective response to their expected return before advancing (e.g., anticipated emotional response if they received their expected return; see Methods). Because Experiments 2 and 3 were nearly exact replications of Experiment 1, we combined the data for all three experiments, in line with recent statistical recommendations^13^ (see Supplemental Material available for results from each individual experiment).

First, we fit a full mixed effects model with drug, self-reported expectations, and their interaction, predicting investment. Because past work has found gender differences in TG-I behavior^14^, we included gender as a covariate. Indeed, replicating past work, there was a main effect of participant gender on the amount of money invested such that women invested significantly less than men (*b* = −0.08 (0.02), *t* = −3.39, *p* < 0.001). More germane to the current focus, there was no main effect of drug on investment but there was a significant interaction between acetaminophen and self-reported expected returns (*b* = −0.16 (0.07), *t* = −2.47, *p* = 0.014; see Fig. S3 and Table S11) such that expectations were less associated with the amounts invested on acetaminophen (*b* = 0.10 (0.05), *t* = 2.23, *p* = 0.027) than on placebo (*b* = 0.26 (0.05), *t* = 5.65, *p* < 0.001). Additional analyses found that there was no effect of drug condition on expectations. Therefore, acetaminophen seemed to influence the correspondence between self-reported expectations and trust behavior but did not modify the expectations.

Based on the results of Experiment 1, we hypothesized that acetaminophen might be blunting the effects of expectations on behavior by reducing affective responses to those expectations. This hypothesis was motivated by previous work showing that acetaminophen reduces the extremity of evaluations^8^. Therefore, in Experiments 2 and 3 we asked participants to evaluate their anticipated affective responses to their expected returns. To analyze this data, we fit a full mixed effects model with drug, self-reported expectations, and their interaction, predicting anticipated affective response to the expected return. The results revealed a nonsignificant interaction between drug condition and expectations (*b* = 0.22 (0.29), *t* = 0.78, *p* = 0.435; see Table S15). Though this interaction was nonsignificant in the combined analyses, it should be noted that this null effect was due to the two underlying experiments producing opposite results (i.e., acetaminophen decreased the relationship between expectations and anticipated affective responses in Experiment 2 but increased it in Experiment 3; see Tables S13 and S14).

#### Trust game as investor; Manipulated expectations

Based on the findings from Experiments 1–3 where acetaminophen seemed to reduce the correspondence between trust game investor expectations and behavior, we conducted two larger lab experiments using another TG-I in which we manipulated expectations about the trustees by providing a single piece of information about their anonymous partner at the beginning of each trial (i.e. “On average, this partner returned ___% of the points they received in past rounds.”). The percentages were derived from actual behavior of trustees in a prior experiment (i.e., Experiment S4) and were selected to range from very untrustworthy (e.g., 0%) to altruistically trustworthy (e.g., 76%). We used actual past trustee behavior from Experiments 1 and 2 to generate the expected return values to ensure that our manipulation spanned the range of observed trustee behaviors from our previous studies. It should be noted though, that the trustees who the investors in Experiments 4 and 5 were paired with were not the trustees whose behavior was used to generate the expected return values. Thus, the manipulation of expectations was false information about the trustees, and participants were informed of this at the end of the experimental session during debriefing. Other than this change, the TG-I in Experiments 4 and 5 were the same as was used Experiments 1–3. Once again, each trial was played with a different trustee and investors did not receive feedback about the trustee decisions.

First, as a manipulation check, we fit a full mixed effects model predicting self-reported expected return with instructed expected return, drug, and their interaction. The results confirmed that the manipulation was successful – instructed expected return significantly predicted self-reported expected return (*b* = 0.17 (0.03), *t* = 5.02, *p* < 0.001). Furthermore, there was no main effect of drug condition on self-reported expected return (*b* = −0.003 (0.02), *t* = −0.17, *p* = 0.87) and no drug by instructed expected return interaction (*b* = −0.03 (0.05), *t* = −0.54, *p* = 0.59). Thus, the manipulation was successful in both drug conditions and there was no effect of drug on overall expectations.

**Figure 2:**
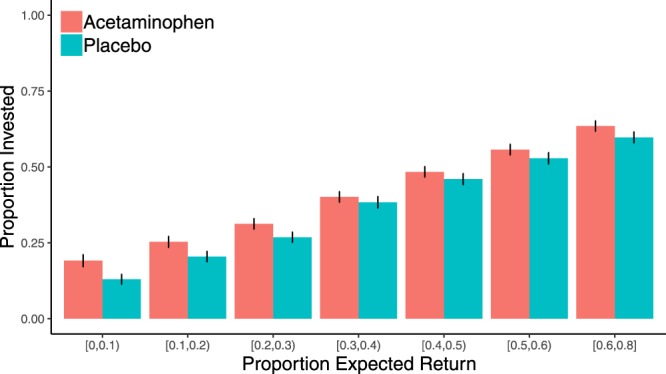
Experiments 4 and 5 combined: Instructed expected return predicting investment. Trials were binned by the expected return that was presented to participants. Because only one trial had an expected return greater than 0.7, this trial was incorporated into the next highest bin in order to the keep the number of trials within each bin roughly equivalent. Participants’ mean investments within each bin was calculated and then the mean of the participant means was plotted. Error bars represent standard error of the mean.

Next, we fit a full mixed effects model with instructed expected return, drug condition, and their interaction, predicting investment. As in Experiments 1–3, the model included participant gender as a covariate. Once again, there was a main effect of gender on investments such that women invested significantly less than men (*b* = −0.09 (0.02), *t* = −4.10, *p* < 0.001). There was a significant effect of instructed expectations on investment (*b* = 0.76 (0.03), *t* = 22.42, *p* < 0.001). Unlike the previous experiments where expectations were not manipulated, there was a marginal main effect of drug (*b* = 0.04 (0.02), *t* = 1.84, *p* = 0.067; see Fig. 2 and Table S22) such that participants on acetaminophen invested more overall. Also contrary to our predictions, there was no drug by instructed expected return interaction on trusting behavior (*b* = −0.03 (0.05), *t* = −0.55, *p* = 0.58) as there had been in Experiments 1–3. Thus, participants who received acetaminophen entrusted more money and did so regardless of whether they had received information indicating that the trustee was likely to betray or reciprocate that trust.

Additional analyses did not find an interaction between drug and self-reported expectations on either investment (*b* = 0.04 (0.16), *t* = 0.26, *p* = 0.79; see Table S23) or anticipated affective response to expected return (*b* = −0.24 (0.38), *t* = −0.62, *p* = 0.54; see Table S24).

#### Additional analyses

We also investigated the breadth of acetaminophen’s effects on social economic behavior by having participants complete other well-validated economic games (see Supplemental Materials available). While we did not find evidence for effects on altruism in a dictator game or generosity among ultimatum game proposers (see Experiments S1 and S2), our results from other social economic paradigms do not necessarily support the idea that acetaminophen’s potential effects are limited to trust. There was preliminary evidence that acetaminophen may reduce the sensitivity of ultimatum game responders to trial-to-trial fluctuations in proposal fairness^15^ (see Experiment S3). There was also some evidence that acetaminophen may reduce the influence of second-order beliefs on reciprocity, potentially due to dampened guilt^16^ (see Experiment S4).

Acetaminophen’s effects on trusting behavior could be due to changes in general risk-taking preferences rather than just social risk. Indeed, there is some work showing that acetaminophen can increase risk-taking^17^. Therefore, the same participants who completed Experiment 2 also completed a nonsocial risk game. Although these same participants showed a reduced relationship between expectations and investments in the TG-I when given acetaminophen, they did not show an effect of acetaminophen on either overall risk-taking or the relationship between expectations and risk (see Experiment S5).

After completing all tasks, participants were asked to guess which drug they received. In all experiments except for 3 and 5, participants were unable to guess which drug they had received (Experiment 1: *χ*^2^ (1, *N* = 111) = 0.01, *p* = 0.91; Experiment 2: *χ*^2^ (1, *N* = 156) = 0.10, *p* = 0.75; Experiment 4: *χ*^2^ (1, *N* = 264) = 0.41, *p* = 0.52). In Experiments 3 and 5, participants guessed at above chance (*χ*^2^ (1, *N* = 211) = 5.30, *p* = 0.021) such that participants who received placebo guessed at above chance (68% correct) while those who received acetaminophen did not (49% correct). Thus, participants were generally unable to guess their drug condition. However, it is worth noting that the same experiments where participants in the placebo condition guessed their condition at above-chance accuracy (i.e., Experiments 3 and 5) were the only two experiments where no drug effects were observed, which may raise the question of whether participants’ beliefs about which drug they received influenced their trust behavior or the effects of acetaminophen. However, including a term for participants’ drug condition guesses revealed no drug guess by drug condition by self-reported expectations in Experiments 1–3 (*b* = −0.09 (0.13), *t* = −0.69, *p* = 0.49) and no drug guess by drug condition interaction in Experiments 4 and 5 (*b* = −0.01 (0.04), *t* = −0.14, *p* = 0.89).

## Discussion

Across an analysis of data from a national survey and five laboratory experiments, we found suggestive evidence that acetaminophen may influence interpersonal trust perceptions and behavior. In a national sample, greater acetaminophen usage was associated with reduced perceptions of trust in one’s neighborhood and reduced feelings of social integration. Additionally, in a series of experimental studies, we found evidence that acetaminophen reduced the influence of self-generated expectations about trustee behavior on investor trusting behavior in a trust game. In large follow-up experiments using a trust game where we manipulated expectations about trustee behavior, investors on acetaminophen entrusted more to their anonymous partners, regardless of expectations.

Our work is the first demonstration that the widely-used, over-the-counter drug acetaminophen may affect the fundamental social process of trust. Thus, the results of our study add to the emerging body of work showing that acetaminophen has psychological and behavioral effects. Though a couple of prior studies have found effects of acetaminophen on decisions, our results go beyond what has been shown before. DeWall and colleagues^19^ suggested that acetaminophen may reduce the endowment effect, but the endowment effect is not typically attributed to social factors to the degree that the trust game is. Instead, it is generally attributed to a differing focus on positive versus negative attributes^20^. Another set of studies found effects of acetaminophen on the severity of hypothetical punishments in response to norm violations^21^. Our findings are largely consistent with this work, but we use real decisions in well-established economic tasks.

Overall, our results suggest possible effects of acetaminophen on trust-related behavior. However, the complexity of our findings suggest that if acetaminophen does indeed influence trust behavior, its effects are unlikely to be straightforward, and critically influenced by situational factors. In what follows, we discuss several of these potential moderating factors.

### Acetaminophen’s Psychological Effects

Although the investigation of acetaminophen’s psychological effects is in its infancy, one mechanism that may account for many of the prior findings is that acetaminophen reduces the extremity of affect and its effects on behavior^5–8,21^. For example, a recent study found that acetaminophen reduced the extremity of participants’ evaluations of both positive and negative emotional images^8^. That study also found that acetaminophen had no effect on the extremity of a non-evaluative judgment (i.e., the amount of blueness in the images)—indicating that acetaminophen’s dampening effect may be selective for affect. Our experiments found mixed support for affective dampening. While acetaminophen reduced the relationship between expectations and anticipated affective responses in Experiment 2, it did the opposite in Experiment 3, which resulted in a null result in the combined analysis. Taken together, these mixed findings suggest that acetaminophen may exert these effects by an affective process not captured by self-report or some non-affective process. Some of our other results are in line with an affective dampening mechanism. Acetaminophen reduced the relationship between counterfactual returns and guilt in trust game trustees^16^. Furthermore, acetaminophen reduced the influence of trial-to-trial fluctuations in fairness on proposal acceptance among ultimatum game responders^15^. Future work should seek alternative mechanisms or draw on the rapidly growing literature on affect and decision-making to clarify when, if, and how acetaminophen’s blunting of affect is likely to influence interpersonal economic decision processes. Consulting these lines of work will be particularly important given the complex nature of affect’s effects on decisions^22–24^.

One psychological mechanism by which acetaminophen could influence trust behavior is by reducing feelings of betrayal aversion^11,25^. Dampened betrayal aversion would produce an overall increase in the amounts invested and thus could account for the effect of acetaminophen in Experiments 4 and 5. Neuroimaging work has also associated anterior insula activity with betrayal aversion^26^ and acetaminophen has been shown to dampen anterior insula responses related to social pain^5^, which lends further support to reduced betrayal aversion as a possible mechanism. However, reduced betrayal aversion cannot account for all of our results. In particular, it does not provide a compelling explanation for the reduced correspondence between expectations and investments observed in Experiments 1–3 because betrayal aversion has generally been observed when controlling for expectations in past studies^27^.

A better understanding of acetaminophen’s psychological effects would also help to improve understanding of the survey data results. One issue is that the survey data is correlational and so it could be that low levels of neighborhood trust lead to greater pain or illness, which brings greater acetaminophen consumption. Though the time period between when the participant took the drug and completed the survey is unclear, the fact that acetaminophen influenced behavioral trust in our placebo-controlled experiments makes it plausible that acetaminophen use could affect perceptions of neighborhood trust. Still, if acetaminophen is indeed influencing perceptions of neighborhood trust, the mechanism remains unclear. Furthermore, it is unclear why there was a negative correlation between acetaminophen usage and neighborhood trust while an acute dose of acetaminophen increased behavioral trust in Experiments 4 and 5. One relevant factor might be age differences between the sample used in the national survey (middle aged and older adults) and the experimental data (young adults). Inflammation increases with age^28^ and because acetaminophen inhibits the brain effects of inflammation^29^ it may have age-dependent effects on trust. Another possibility is that while our laboratory experiments used an acute dose of acetaminophen, the survey measured chronic usage. Chronic acetaminophen use may produce neural adaptations such as alterations in receptor density. It is then these receptor changes that could be responsible for the reduced neighborhood trust. A third possible explanation stems from the fact that our experimental results found that acutely administered acetaminophen increased trust behavior towards partners who were explicitly expected to betray that trust. Regular acetaminophen consumption may therefore increase the frequency with which people misplace trust in others whom they would otherwise avoid. Increased rates of misplaced trust would thus also increase their experiences of betrayal, which may ultimately lead to the development of more distrustful perceptions of the world. A longitudinal experiment examining effects of chronic acetaminophen consumption on social behavior and social perceptions would shed light on these and other possible explanations.

### Reputational Knowledge about Partner and Differential Effects of Acetaminophen

An intriguing result of these studies is that acetaminophen had different effects depending on the implementation of the TG-I. While acetaminophen increased trusting behavior overall when participants were provided with expectations for trustee behavior, it did not have this effect when participants were required to make their decisions without any information about their partner. Consistent with the different pharmacological effects in these different versions of the TG-I, prior neuroimaging results suggest that the neural processes involved in making trust decisions vary across instantiations of the TG-I. For example, an fMRI study found that the anterior insula showed greater involvement during trust decisions when lacking prior information about the reputation of a trustee than when information was not provided^30^. This is in line with a recent meta-analysis that found greater anterior insula involvement during one-shot TG-I games, where there is a lack of knowledge about the partner, relative to multiround trust games, where the trustor plays with the same partner over multiple trials^31^. Experiments 1–3 of the present study were one-shot decisions without any information about past behavior. In contrast, because the investors in Experiments 4 and 5 formed expectations about each trustee’s reciprocity based on information about past behavior, these experiments bear some similarity to multiround trust games. The same meta-analysis did not find any overlap in neural activations between one-shot and multiround trust games, indicating that it is feasible for a drug such as acetaminophen to have differential effects in separate instantiations of the TG-I due to the different neural circuits involved.

These neural differences across instantiations of the TG-I suggest that trust decisions made in different contexts may rely on different psychological processes that could be differentially impacted by acetaminophen^31^. Although a few studies have examined differences in trusting behavior across repeated interactions when investors were either given prior expectations or not^30,32^, more research is needed to understand how trust decisions differ psychologically when information predicting reciprocity is or is not available. One possible difference is that when participants are not provided with any expectations, they must generate them on their own, presumably by drawing on past experiences with other people in similar situations. This process is likely not required when participants are provided with expectations. In this way, our two trust games parallel the distinction between decision-making under risk versus ambiguity, which have also been shown to rely on different neural processes^33,34^. That is, Experiments 1–3 reflect decisions made under social ambiguity while Experiments 4 and 5 entail decisions made under social risk. In this way, acetaminophen might be expected to interact with expectations to impact trust in socially ambiguous situations but to affect trust behavior directly when the social risk is known. Future work should examine trust decisions made in different contexts and consider the processes involved in order to make predictions about what sorts of effects acetaminophen might have.

### Broader Implications

Though the effects found in our lab experiments were small, a relatively minor change in a person’s behavior can have an amplified impact in the context of more dynamic and repeated social interactions (e.g., see ref.^35^). This is especially likely to be the case with alterations to a fundamental social process such as trust, which is important across a wide-range of social contexts^36^ and is critical to the development and maintenance of relationships^37,38^. However, these pharmacological effects could also have implications that extend beyond interpersonal relationships. For example, less trust is associated with myopic economic choices and boosting community-level trust can reduce the discounting of future economic outcomes observed among people with low incomes^39^. At a societal level, the amount of interpersonal trust among a country’s population is predictive of future economic growth^3^ and is associated with increased participation in civic activities and decreased government corruption^4^. Thus, pharmacological alterations to trust may trigger reverberations that are felt throughout a social system.

## Methods

### Participants

Participants were undergraduate students (Experiment 1: 73 males, 45 females, 4 unknown; Experiment 2: 60 males, 95 females, 7 unknown; Experiments 3 & 5: 106 males, 103 females, 3 unknown; Experiment 4: 148 males, 123 females) (see description of participant exclusions below). The sample sizes for Experiment 1 was based on the effect size observed in another set of acetaminophen studies (Cohen’s *d* = 0.55)^8^. Experiment 2 was conducted later and used a more conservative effect size that was observed in a recent set of acetaminophen studies (Hedge’s *g* = 0.45)^7^. For Experiments 3, 4, and 5, we aimed for larger sample sizes and continued data collection until the end of the semester. All participants in Experiment 2 received either course credit or a base payment as compensation for showing up. In all other experiments, participants received course credit for showing up. Across all lab experiments, participants were additionally paid based on one randomly selected decision. Each participant was randomly paired with an anonymous participant from a separate study for one decision and each was paid according to the decisions made by each player. Payments earned based on the randomly selected decision ranged from $0 to $10.

For all experiments except Experiment 1, participants completed an initial general quiz over instructions about the experiment (e.g., whether they would be playing the economic games with other participants or the computer) as well as a quiz later on about the specific task (e.g., how much investments would be multiplied by) (see Supplemental Material available for quiz questions). There was no quiz in Experiment 1. Each quiz question had 3 multiple-choice response options. Although the quizzes gave real-time feedback and required participants to answer all questions correctly before advancing, some participants did not perform above chance level, which suggests a definite lack of comprehension/motivation. Therefore, we excluded participants who did not perform significantly above chance on either quiz (i.e., <0.4 chance of getting answers correct). We calculated guessing accuracy by dividing the number of questions in a quiz by the total number of responses given before passing the quiz. Note that because participants received real-time feedback that their answers were incorrect, our cutoff criterion for exclusion is quite lenient. Although poor task comprehension is a strong indicator that these participants’ data would not be informative of the psychological and social processes that we aimed to study, we also report the results of our analyses without exclusions in the interest of transparency (see Supplemental Material available). Six participants were excluded from Experiment 2, 5 participants were excluded from Experiment 3, 5 participants were excluded from Experiment 4, and 7 participants were excluded from Experiment 5.

### General Procedures

The OSU Institutional Review Board approved the experiments and they were conducted in accordance with its guidelines. All experiments followed the same general procedure. After signing up for the experiments, participants received an email informing them about the risk factors associated with acetaminophen (e.g., currently taking a drug containing acetaminophen, a history of liver disorder, an allergic reaction to acetaminophen, currently taking an anticoagulant, or a history of alcohol abuse) and asked them to refrain from participation if they met any of these risk factors. To facilitate drug absorption, we also asked participants to refrain from consuming food for three hours before the experiment.

Upon arrival, all participants provided informed consent and then were randomly assigned to take either an acute dose of 1,000 mg of acetaminophen or a placebo, both in a liquid vehicle (Experiment 1: 61 acetaminophen, 61 placebo; Experiment 2: 81 acetaminophen, 81 placebo; Experiments 3 & 5: 105 acetaminophen, 107 placebo; Experiment 4: 138 acetaminophen, 133 placebo). Acetaminophen and placebo solutions were prepared by Pharmacy Specialists Compounding Pharmacy (Altamonte Springs, Florida; http://www.makerx.com/). The drug solution consisted of acetaminophen (100 mg/ml) dissolved in Ora-Plus suspension liquid and flavored with Ora-Sweet Syrup. The placebo solution consisted of Avicel Microcrystalline powder (100 mg/ml) dissolved in the same vehicle. Assignment of drug condition followed a double-blind procedure such that both experimenters and participants were unaware of participants’ assigned conditions. Bottles containing the drug and placebo mixtures were labeled with codes and participants were randomly assigned to condition upon arrival. Drug condition was decoded after data collection was completed.

After receiving the drug, participants completed self-report questionnaires and then were allowed to rest until approximately 60 minutes had elapsed. This uptake period was to allow sufficient time for the drug to enter the brain^18^. Then participants completed a trust game in the role of investor (TG-I). Some participants also completed a dictator game, ultimatum game in the role of either proposer or responder, or trust game in the role of trustee (see Supplemental Material available for methods and results). All tasks were completed as part of larger social and cognitive assessments (to be reported separately). Participants completed the self-report questionnaires and tasks within individual cubicles. After completing all tasks, participants were asked to guess which drug they received.

### Experiments 1–3: Trust Game as Investor with No Information on Partner's Past Behavior

The TG-I used in Experiments 1–3 were nearly identical and based on the design by Chang and colleagues^16^. Any differences are noted in the following description. In Experiment 1, participants were given initial endowments of 4 different sizes in a fixed order whereas participants in Experiment 2 and 3 were given initial endowments of 6 different sizes, which were randomly ordered for each participant. For each trial in both studies, participants were asked to decide how much, if any, of the endowment they wished to send to a partner who they were informed would be another participant taking part in a similar experiment. Participants were informed that all players in the games would be anonymous. The amount sent to the partner (i.e. the trustee) was multiplied times 4 and participants were instructed that their partner would decide how much of this new amount to keep for him/herself and how much to return to the participant. The trustee decisions occurred in a separate experiment, and thus investors did not receive any feedback about trustee behavior during the task. After each investment decision, participants were asked how much they expected their partner to return to them, again with an open response. On trials where participants did not send anything to their partner, they were not asked how much they expected to have returned. On each trial in Experiments 2 and 3, participants were then asked three questions in reference to their reported expected return. These questions were intended to assess affective responses to their expectations and mirrored the exact wording of a prior acetaminophen study on affect^8^. The first question measured evaluation and asked “If your partner returned [expected return] points, to what extent would this outcome be positive or negative?”. Responses were given with an 11-point scale ranging from “extremely negative” (−5) to “extremely positive” (+5). Next, emotional arousal was measured by asking participants “If your partner returned [expected return] points, to what extent would this outcome make you feel an emotional reaction?”. Responses were given with an 11-point scale ranging from “not at all” (0) to “extremely” (10). Finally, participants were asked “If your partner returned [expected return] points, how would this make you feel?” and gave their response using the emoticons from the self-assessment manikin (SAM)^40^, which features 9 faces ranging from sad to happy in expression. The evaluation and SAM items were highly correlated (*r*(2075) = 0.71, *p* < 0.001) and therefore each measure was z-scored and then the two items were averaged to form a general evaluative composite.

For analyses, the proportion of the initial endowment that was invested as well as the proportion of the amount the trustee received that the investor expected to have returned were calculated for each trial. In all TG-I analyses that included expectations, trials on which participants did not invest anything were excluded because the participants could not be asked for their expectations (4.7%, 2.6%, and 5.2% of trials in Experiments 1, 2, and 3, respectively). However, another set of analyses in which expectations for these trials were inferred to be zero did not change any of the results. When testing whether drug condition had an effect on how much of their investments participants expected to have returned, each participants’ expectations were averaged.

### Experiments 4 and 5: Trust Game as Investor with Manipulated Expectations

The TG-I used in Experiments 4 and 5 were highly similar to the ones used in Experiments 1–3. For this task, participants were given the same size endowment for each of 30 trials in Experiment 4. For Experiment 5, the task was shortened to 15 trials because analyses of Experiment 4 showed the effect remained unchanged when the number of trials analyzed was reduced. On each trial, participants were asked to decide how much, if any, of the endowment they wished to send to a partner who they were informed would be another participant taking part in a similar experiment. Participants were informed that all players in the games would be anonymous. This time though, each trial also included the following message on the investment screen: “On average, this partner returned ___% of the points they received in past rounds.” Different amounts ranging from 0 to 76 filled the blank on each trial in random order. These amounts were derived from the actual behavior of past trustees and were selected to follow a roughly uniform distribution. Participants were told that their partners had completed the same task in the past and that these numbers reflected their actual behavior. As in Experiments 1–3, the amount the sent to the partner (i.e. the trustee) was multiplied times 4 and participants were instructed that their partner would decide how much of this new amount to keep for him/herself and how much to return to the participant. After every fifth investment decision (i.e., trials 5, 10, etc.), participants were asked how much they expected their partner to return to them, again with an open response. If participants did not send anything to their partner on this trial, the question was skipped. On these same trials, participants were then asked the same evaluation and feeling questions in reference to their reported expected return as in Experiments 2 and 3. The first question measured evaluation and asked “If your partner returned [expected return] points, to what extent would this outcome be positive or negative?”. Responses were given with an 11-point scale ranging from “extremely negative” (−5) to “extremely positive” (+5). Next, participants were asked “If your partner returned [expected return] points, how would this make you feel?” and gave their response using the emoticons from the self-assessment manikin (SAM)^40^, which features 9 faces ranging from sad to happy in expression. The arousal question was not included. Participants did not receive any feedback on their decisions as a part of the task. The participants who completed Experiment 5 were the same participants who completed Experiment 3 and always completed both tasks in the same experimental session with the TG-I with manipulated expectations coming second.

### National Survey Data

The National Survey of Midlife Development in the United States (MIDUS II) was a longitudinal follow up of the original MIDUS I study, which was conducted in 1995 and 1996. Participants completed measures from project 1 of the MIDUS-II in 2004–2006.

#### Acetaminophen Usage

A subset of participants (N = 3,410) responded to a question asking if they used nonprescription drugs containing acetaminophen in the past 30 days (Yes or no). If they selected “yes”, participants were asked to indicate how frequently they have used acetaminophen in the past 30 days (daily, a few times a week, once a week, a few times a month, once this month). These responses were used to rank participants into 6 categories of acetaminophen usage frequency (0 = not at all [N = 1,981], 1 = Once this month [N = 298], 2 = A few times this month [N = 501], 3 = Once A week [N = 165], 4 = A few times a week [N = 307], 5 = Daily [N = 158]).

#### Perceived neighborhood trust

Participants completed a 4-item scale assessing perceptions of neighborhood trust and safety^9^. Responses were given on a 4-point scale (1 = a lot, 4 = not at all) and this was reverse scored so that high scores reflect high perceived neighborhood trust (α = 0.64).

#### Social integration

Participants completed a 3-item scale assessing feelings of social integration with their community^9^. Responses were given on a 7-point scale (1 = Strongly agree, 7 = Strongly disagree). The measure was scored so that high scores reflect stronger feelings of social integration (α = 0.75).

#### Covariates

We controlled for participant sex, age, education, and overall household income. Because many individuals take acetaminophen to relieve pain, we included the experience of chronic pain as a covariate in all analyses. Chronic pain was measured by participants indicating whether they had “pain that persisted beyond the time of normal healing and has lasted anywhere from a few months to many years” (yes or no).

### Data Analysis

All analyses were conducted in the R statistical package^41^.

#### Lab experiments

For regressions that included repeated observations, linear mixed effects models were fit to the data. Mixed effects models were fit with the R package lme4^42^. P-values were obtained from the lmerTest package^43^. Drug condition was dummy coded (Acetaminophen = 1; Placebo = 0). Continuous predictors were analyzed both as grand mean centered and with the between- and within-participant variance separated (the former is reported in the main text whereas the latter is available in the Supplemental Material).

#### National survey data

All continuous variables were standardized. Dichotomous variables were dummy-coded (i.e., chronic pain: 1 = yes, 0 = no; sex: 1 = male, 0 = female).

Because of high levels of skewness in the variables, regression analysis using bootstrapping was used for all analyses. Bootstrapping is a nonparametric statistical resampling procedure can be used when the distributional assumptions of parametric analyses are not met. All bootstrapped regressions used 5,000 resampled iterations to arrive at a 95% bias corrected and accelerated confidence intervals for regression coefficients. Significant effects are indicated by confidence intervals that do not include 0.

## Supplementary information

Supplementary Materials

## Data Availability

The data that support the findings of this study are available on request from the corresponding author (IDR). The data are not publicly available due to a restriction in the consent forms stating that data would not be shared publicly.
