# Supplementary material for: Acetaminophen influences social and economic trust
Source: Sci Rep. 2019 Mar 11;9:4060. doi: 10.1038/s41598-019-40093-9 (PMC6412049; doi:10.1038/s41598-019-40093-9)
Supplement: Supplementary file 1 — Supplementary Materials [file 41598_2019_40093_MOESM1_ESM.docx]

Acetaminophen influences social and economic trust

Ian D. Roberts^a,1^, Ian Krajbich^b,c, *^, and Baldwin M. Way^b,d,1,*^

^a^Department of Psychology, University of Toronto, 1265 Military Trail, Toronto, Ontario M1C 1A4, Canada

^b^Department of Psychology, The Ohio State University, 1835 Neil Avenue, Columbus, OH 43210, USA

^c^Department of Economics, The Ohio State University, 1945 N High Street, Columbus, OH 43210, USA

^d^Institute for Behavioral Medicine Research, The Ohio State University, 460 Medical Center Drive, Columbus, OH 43210, USA

^1^To whom correspondence should be addressed. Email to iandavidroberts@gmail.com

*denotes joint last authors

# Supplementary Materials

[Supplementary Materials 1](#_Toc525971900)

[Further Description of Survey Data Analyses 4](#_Toc525971901)

[Additional Plots 6](#_Toc525971902)

[Figure S1. Survey data results for ibuprofen 6](#_Toc525971903)

[Figure S2. Survey data results for aspirin 7](#_Toc525971904)

[Figure S3. Experiments 1-3: Expected return predicting investment 8](#_Toc525971905)

[Figure S4. Experiments 2-3: Expected return predicting investment with median split by weight 9](#_Toc525971906)

[Figure S5. Experiments 4-5: Instructed expected return predicting investment with median split by weight 10](#_Toc525971907)

[Figure S6: Experiment S3: Effect of change in proposal fairness on proposal acceptance 11](#_Toc525971908)

[Figure S7. Experiment S4: Second-order belief predicting actual amount returned 12](#_Toc525971909)

[Figure S8. Experiment S4: Counterfactual return predicting counterfactual guilt 13](#_Toc525971910)

[Additional Experiments 14](#_Toc525971911)

[Experiment S1: Dictator Game 14](#_Toc525971912)

[Experiment S2: Ultimatum Game as Proposer 14](#_Toc525971913)

[Experiment S3: Ultimatum Game as Responder 15](#_Toc525971914)

[Experiment S4: Trust Game as Trustee 17](#_Toc525971915)

[Experiment S5: Risk Game 19](#_Toc525971916)

[Tables 21](#_Toc525971917)

[Table S1. Descriptive Statistics 21](#_Toc525971918)

[Table S2. Survey Data: Acetaminophen Usage Predicting Neighborhood Trust 22](#_Toc525971919)

[Table S3. Survey Data: Acetaminophen Usage Predicting Social Integration 23](#_Toc525971920)

[Table S4. Survey Data: Ibuprofen Usage Predicting Neighborhood Trust 24](#_Toc525971921)

[Table S5. Survey Data: Ibuprofen Usage Predicting Social Integration 25](#_Toc525971922)

[Table S6. Survey Data: Aspirin Usage Predicting Neighborhood Trust 26](#_Toc525971923)

[Table S7. Survey Data: Aspirin Usage Predicting Social Integration 27](#_Toc525971924)

[Table S8. Experiment 1: Drug X Expectations on Investment 28](#_Toc525971925)

[Table S9. Experiment 2: Drug X Expectations on Investment 29](#_Toc525971926)

[Table S10. Experiment 3: Drug X Expectations on Investment 30](#_Toc525971927)

[Table S11. Experiments 1, 2, & 3 Combined: Drug X Expectations on Investment 31](#_Toc525971928)

[Table S12. Experiments 1, 2, & 3 Combined: Drug on Expected Return 32](#_Toc525971929)

[Table S13. Experiment 2: Effect of Drug on Anticipated Affective Responses to Expected Returns 33](#_Toc525971930)

[Table S14. Experiment 3: Effect of Drug on Anticipated Affective Responses to Expected Returns 34](#_Toc525971931)

[Table S15. Experiments 2 & 3 Combined: Effect of Drug on Anticipated Affective Responses to Expected Returns 35](#_Toc525971932)

[Table S16. Experiments 2 & 3 Combined: Effect of Drug on Anticipated Emotional Arousal to Expected Returns 36](#_Toc525971933)

[Table S17. Experiments 2 & 3 Combined: Drug X Expectations X Weight (kg) on Investment 37](#_Toc525971934)

[Table S18. Experiments 2 & 3 Combined: Drug X Expectations on Investment (Low Weight) 38](#_Toc525971935)

[Table S19. Experiments 2 & 3 Combined: Drug X Expectations on Investment (High Weight) 38](#_Toc525971936)

[Table S20. Experiment 4: Drug X Instructed Expectations on Investment 39](#_Toc525971937)

[Table S21. Experiment 5: Drug X Instructed Expectations on Investment 40](#_Toc525971938)

[Table S22. Experiments 4 & 5 Combined: Drug X Instructed Expectations on Investment 41](#_Toc525971939)

[Table S23. Experiments 4 & 5 Combined: Drug X Self-Reported Expectations on Investment 42](#_Toc525971940)

[Table S24. Experiments 4 & 5 Combined: Effect of Drug on Anticipated Affective Responses to Self-Reported Expected Returns 43](#_Toc525971941)

[Table S25. Experiments 4 & 5 Combined: Drug X Instructed Expectations X Weight on Investment 44](#_Toc525971942)

[Table S26. Experiments 4 & 5 Combined: Drug X Instructed Expectations on Investment (Low Weight) 45](#_Toc525971943)

[Table S27. Experiments 4 & 5 Combined: Drug X Instructed Expectations on Investment (High Weight) 45](#_Toc525971944)

[Table S28. Experiment S3: Drug X Proposal Fairness on Proposal Acceptance 46](#_Toc525971945)

[Table S29. Experiment S3: Drug X Change in Proposal Fairness on Proposal Acceptance 47](#_Toc525971946)

[Table S30. Experiment S4: Drug X Second-Order Beliefs on Amount Returned 48](#_Toc525971947)

[Table S31. Experiment S4: Drug X Counterfactual Return on Counterfactual Guilt 49](#_Toc525971948)

[Table S32. Experiment S5: Drug X Expectations on Bet 50](#_Toc525971949)

[Table S33. Experiment S5: Drug X Expectations on Anticipated Affective Responses to Expected Win 51](#_Toc525971950)

[Task Instructions and Quizzes 52](#_Toc525971951)

[Experiments 1, S1, & S2: General Instructions 52](#_Toc525971952)

[Experiment S1: Dictator Game Instructions 53](#_Toc525971953)

[Experiment 1: Trust Game as Investor Instructions 54](#_Toc525971954)

[Experiment S2: Ultimatum Game as Proposer Instructions 55](#_Toc525971955)

[Experiment 2: General Instructions 56](#_Toc525971956)

[Experiment 2: General Instructions Quiz 57](#_Toc525971957)

[Experiment 2: Trust Game as Investor Instructions 58](#_Toc525971958)

[Experiment 2: Trust Game as Investor Quiz 59](#_Toc525971959)

[Experiment 3: Instructions 60](#_Toc525971960)

[Experiment 3: Instructions Quiz 61](#_Toc525971961)

[Experiment 4: General Instructions 63](#_Toc525971962)

[Experiment 4: General Instructions Quiz 64](#_Toc525971963)

[Experiment 4: Trust Game as Investor Instructions 65](#_Toc525971964)

[Experiment 4: Trust Game as Investor Quiz 66](#_Toc525971965)

[Experiment 5: Instructions 67](#_Toc525971966)

[Experiment 5: Instructions Quiz 68](#_Toc525971967)

[Experiments S3 & S4: General Instructions 70](#_Toc525971968)

[Experiments S3 & S4: General Instructions Quiz 71](#_Toc525971969)

[Experiment S3: Ultimatum Game Instructions 72](#_Toc525971970)

[Experiment S3: Ultimatum Game Quiz 73](#_Toc525971971)

[Experiment S4: Trust Game as Trustee Instructions 74](#_Toc525971972)

[Experiment S4: Trust Game as Trustee Quiz 75](#_Toc525971973)

[Experiment S5: Risk Game Instructions 76](#_Toc525971974)

[Experiment S5: Risk Game Quiz 76](#_Toc525971975)

[Results without Participant Exclusions 78](#_Toc525971976)

[Experiment 2 (TG-I). 78](#_Toc525971977)

[Experiment 3 (TG-I). 78](#_Toc525971978)

[Experiment 4 (TG-I with manipulated expectations). 78](#_Toc525971979)

[Experiment 5 (TG-I with manipulated expectations). 78](#_Toc525971980)

[Experiment S3 (UG-R). 78](#_Toc525971981)

[Experiment S4 (TG-T). 79](#_Toc525971982)

[References 80](#_Toc525971983)

# Further Description of Survey Data Analyses

In addition to our analyses using acetaminophen usage as a predictor, we also conducted similar analyses with ibuprofen and aspirin usage as predictors instead. Methods and results are reported below.

*Ibuprofen Usage.* A subset of participants (N = 3,414) responded to a question asking if they used nonprescription drugs containing ibuprofen in the past 30 days (Yes or no). If they selected “yes”, participants were asked to indicate how frequently they have used ibuprofen in the past 30 days (daily, a few times a week, once a week, a few times a month, once this month). These responses were used to rank participants into 6 categories of ibuprofen usage frequency (0 = not at all [N = 1,862], 1 = Once this month [N = 299], 2 = A few times this month [N = 591], 3 = Once A week [N = 172], 4 = A few times a week [N = 358], 5 = Daily [N = 132]).

*Aspirin Usage.* A subset of participants (N = 3,603) responded to a question asking if they used nonprescription drugs containing aspirin in the past 30 days (Yes or no). If they selected “yes”, participants were asked to indicate how frequently they have used aspirin in the past 30 days (daily, a few times a week, once a week, a few times a month, once this month). Participants reported whether they took a regular dose of aspirin for a heart condition. Because doses of aspirin for heart conditions are typically much lower than the usual dose taken for pain relief, we excluded these participants (N = 1,527). These responses were used to rank participants into 6 categories of aspirin usage frequency (0 = not at all [N = 1,489], 1 = Once this month [N = 184], 2 = A few times this month [N = 298], 3 = Once A week [N = 83], 4 = A few times a week [N = 191], 5 = Daily [N = 110]).

*Additional Covariates.* In addition to controlling for participant sex, age, education, overall household income, and chronic pain, we also ran models controlling for some additional variables. Because number of years living in a neighborhood is likely to influence feelings of neighborhood trust and social integration, we also controlled for this. Length of time living in the current neighborhood was measured with the following question: “How many years have you lived in your current neighborhood, or if you live in a rural area, in your current township? (If less than one year, enter ‘0’.)” Also, because poor physical or mental/emotional health could lead to social withdrawal (and thus, reduced social integration) or to self-medicating with pain-killers, we controlled for these variables as well. Physical health was measured with the question “In general, would you say your PHYSICAL HEALTH is excellent, very good, good, fair, or poor?” Mental/emotional health was measured with the question “What about your MENTAL OR EMOTIONAL HEALTH? (Would you say your MENTAL OR EMOTIONAL HEALTH is excellent, very good, good, fair, or poor?)”

# Additional Plots

## Figure S1. Survey data results for ibuprofen


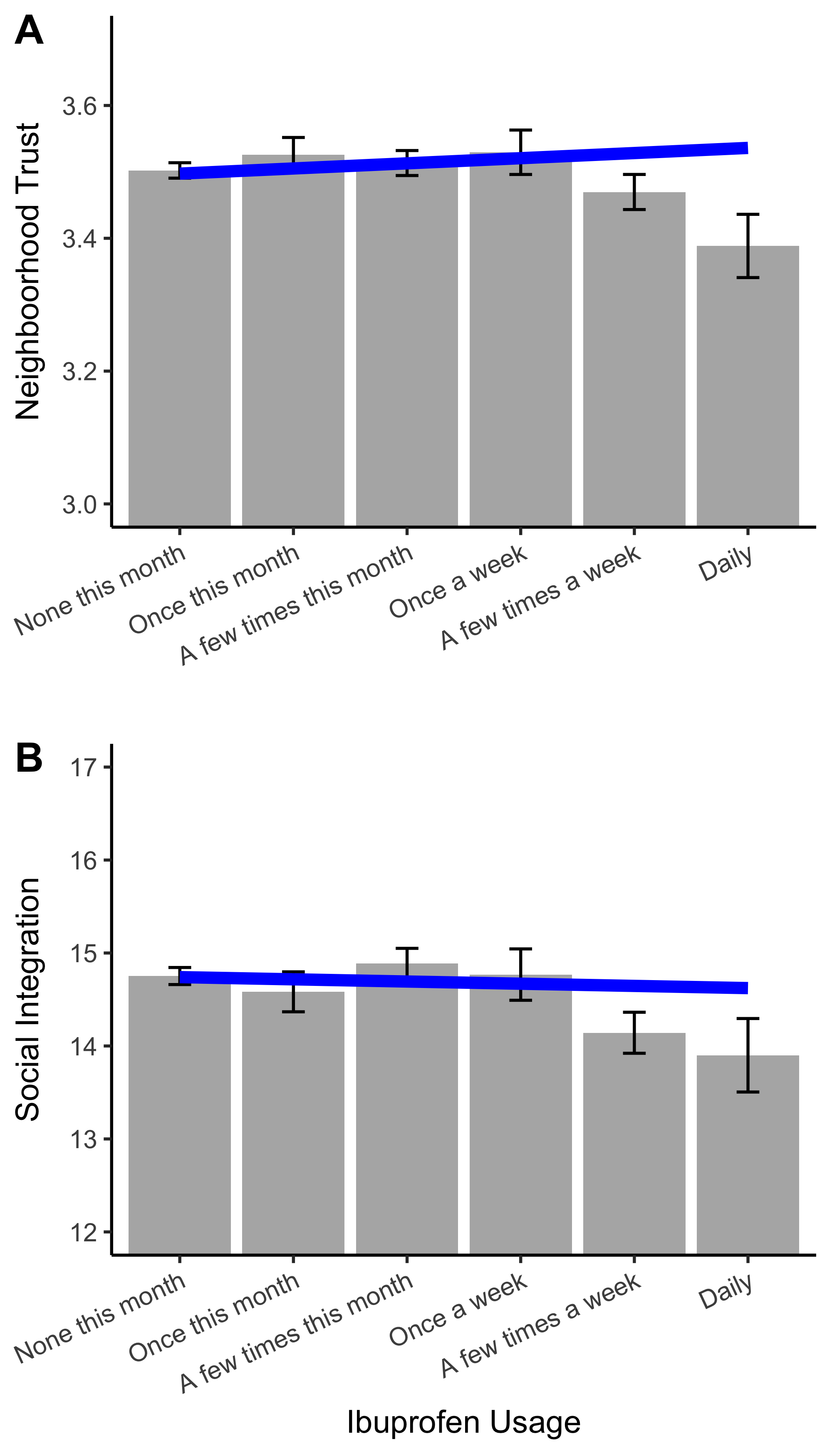


## Figure S2. Survey data results for aspirin


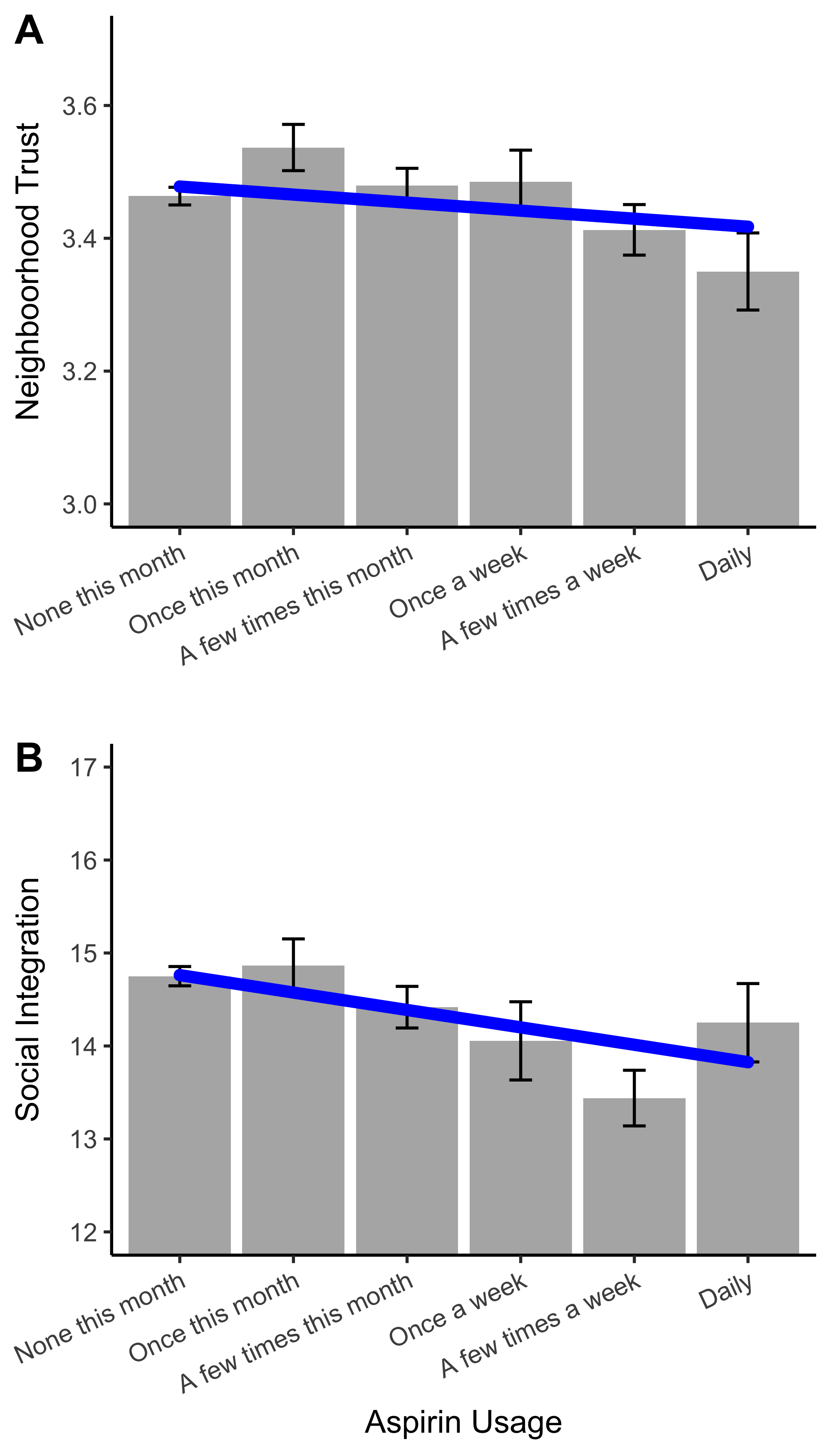


## Figure S3. Experiments 1-3: Expected return predicting investment


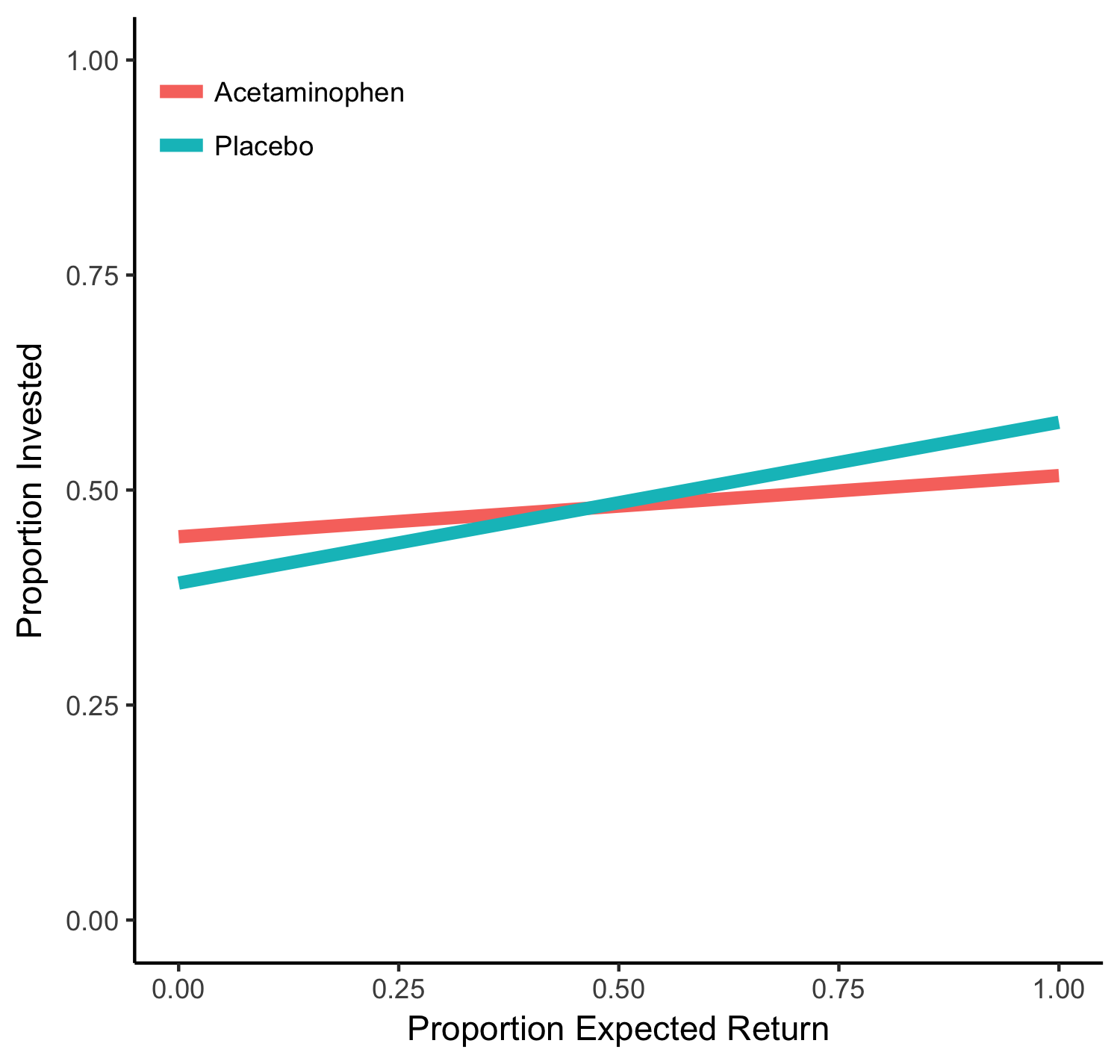


Experiments 1-3 combined: expected return predicting investment. Predicted values from the model displayed in Table S11.

## Figure S4. Experiments 2-3: Expected return predicting investment with median split by weight


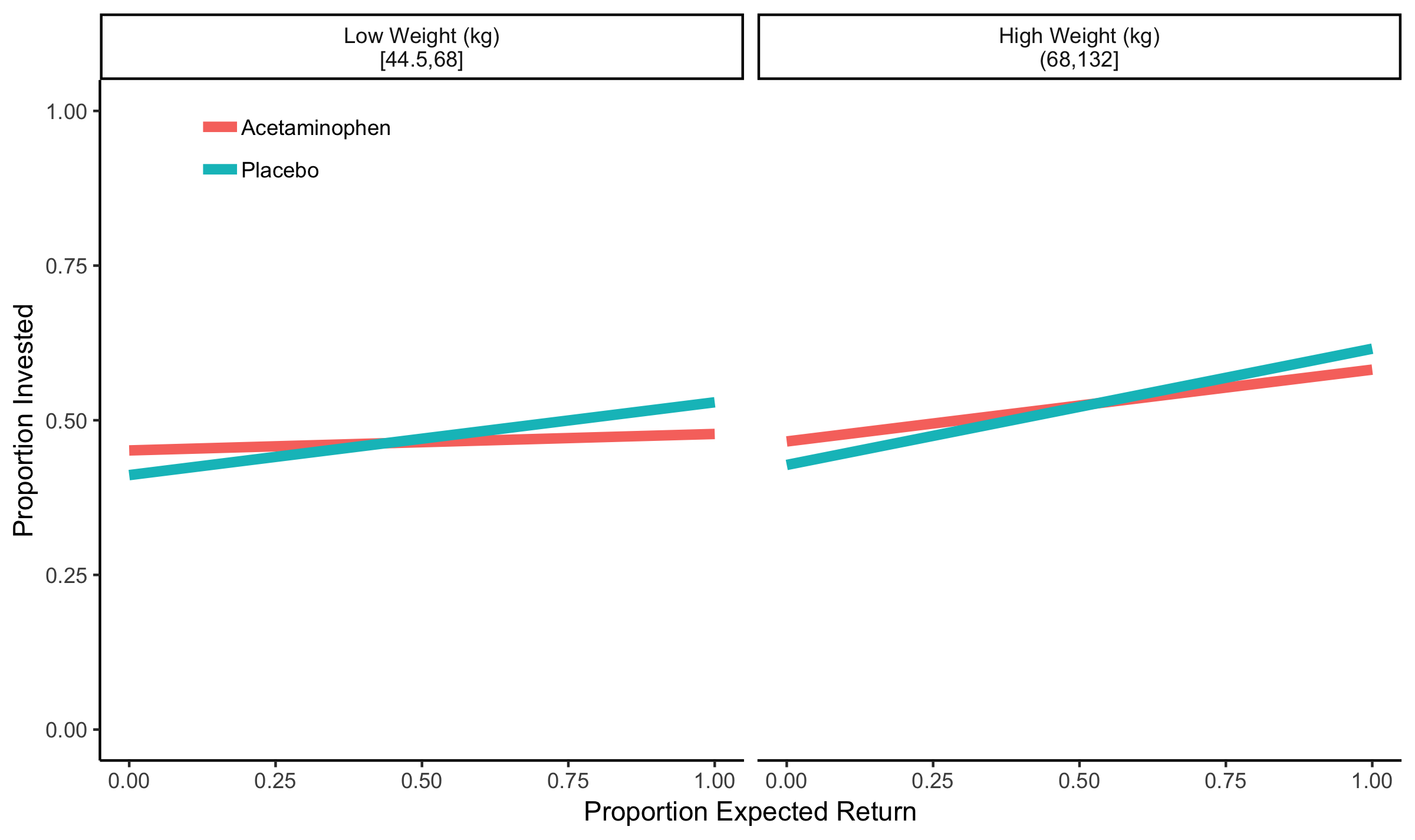


Experiments 2 and 3 combined with a median split by participant weight: expected return predicting investment. Participant weight was not collected in Experiment 1 and so data from that experiment could not be included in this analysis. Predicted values are from the models displayed in Tables S18 & S19.

## Figure S5. Experiments 4-5: Instructed expected return predicting investment with median split by weight

*
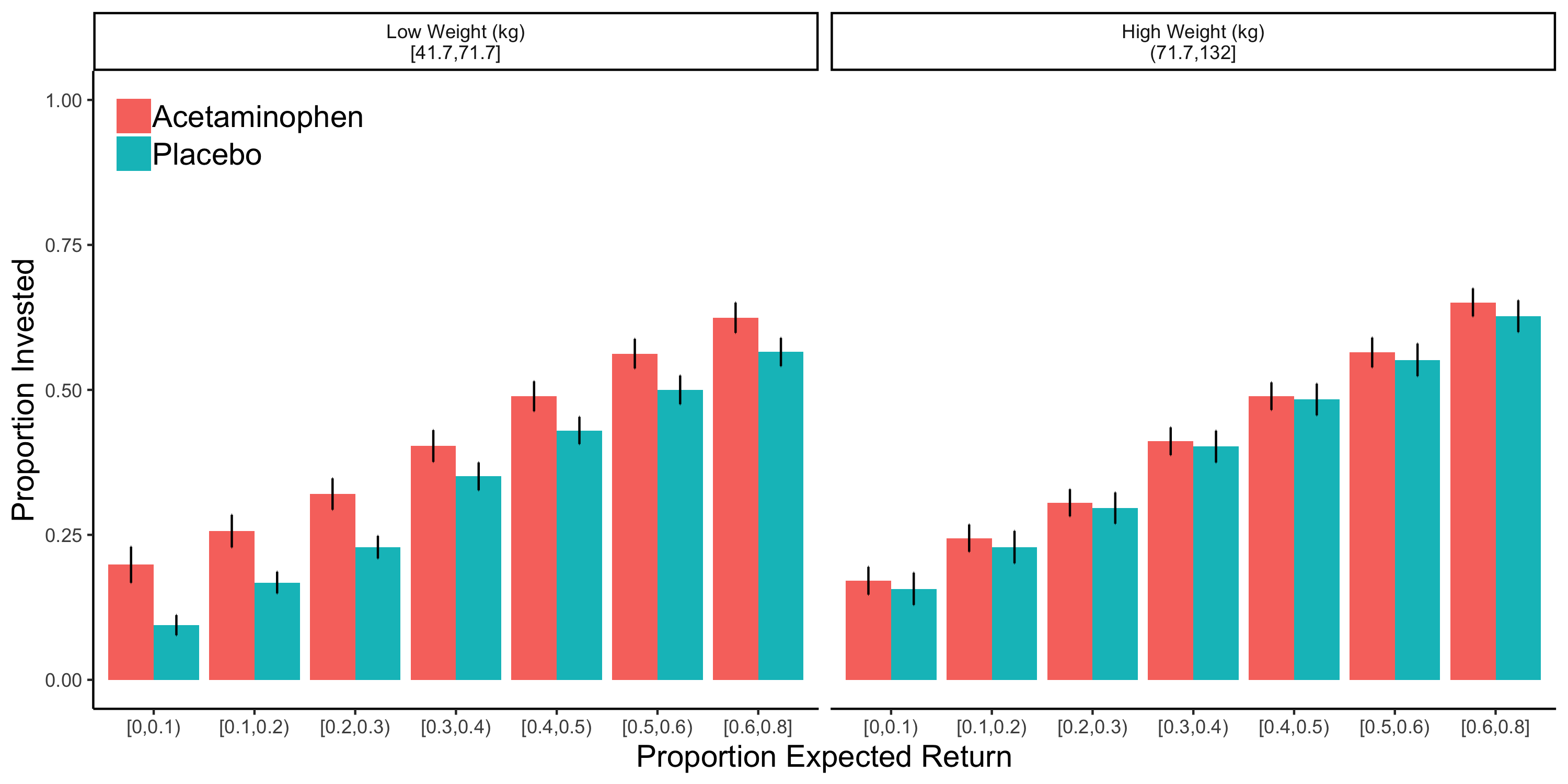
*

Experiments 4 and 5 combined with a median split by participant weight: Instructed expected return predicting investment. Trials were binned by expected return presented to participants. Because only one trial had an expected return greater than 0.7, this trial was incorporated into the next highest bin in order to the keep the number of trials within each bin roughly equivalent. Participants’ mean investments within each bin was calculated and then the mean of the participant means was plotted. Error bars represent standard error of the mean. Statistics reported in Tables S25-S27.

## Figure S6: Experiment S3: Effect of change in proposal fairness on proposal acceptance


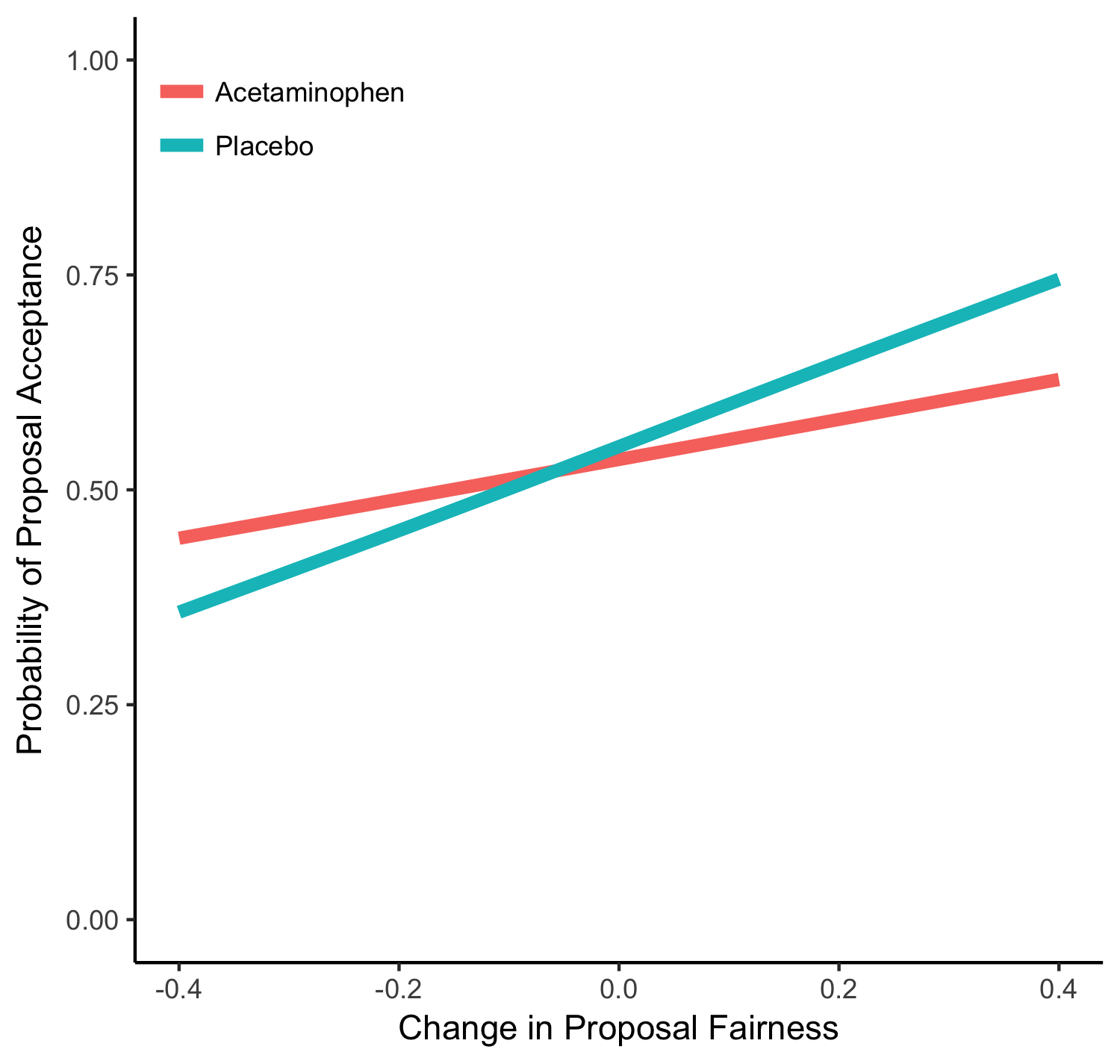


Experiment S3: Change in offer fairness relative to previous trial predicting probability of offer acceptance. Predicted values from model displayed in Table S23.

## Figure S7. Experiment S4: Second-order belief predicting actual amount returned


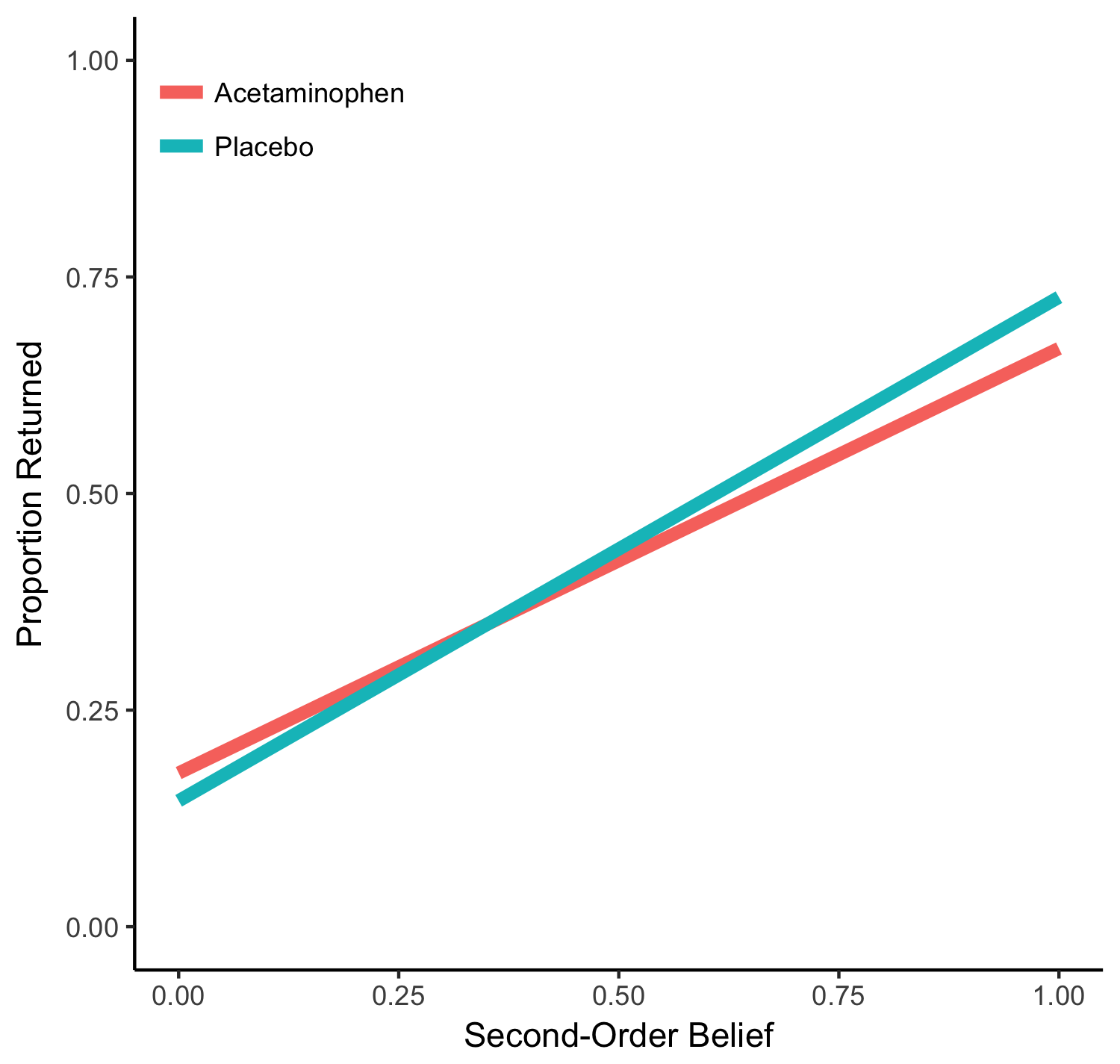


Experiment S4: Second-order beliefs predicting actual amount returned. Predicted values from model displayed in Table S24.

## Figure S8. Experiment S4: Counterfactual return predicting counterfactual guilt


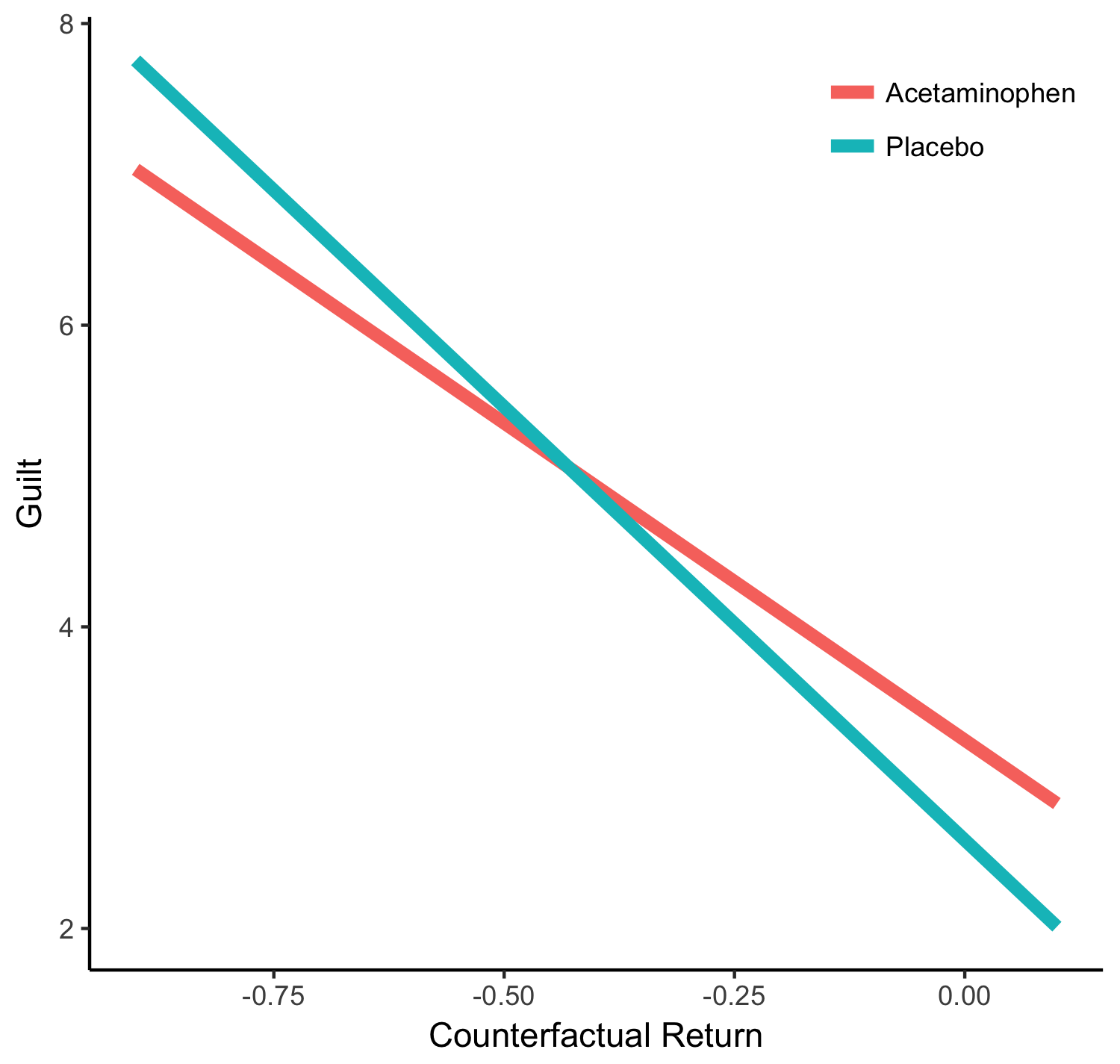


Experiment S4: Difference between the counterfactual return (i.e., the alternative hypothetical return that was presented) and their actual return predicting guilt. Predicted values from model displayed in Table S26.

# Additional Experiments

## Experiment S1: Dictator Game

The same participants who completed the TG-I in Experiment 1 also completed a dictator game^1^. In the dictator game, participants were given initial endowments of 4 different sizes in a fixed order. For each trial, participants were asked to decide how much, if any, of the endowment they wished to give to an anonymous partner who they were informed would be another participant taking part in a similar experiment.

We fit a mixed effects model with drug and gender predicting the proportion of the endowment that was given. The intercept was allowed to vary randomly for participants. There were no effects of either gender (*b* = -0.05 (0.04), *t* = -1.24, *p* = .22) or drug condition (*b* = 0.02 (0.04), *t* = 0.47, *p* = .64).

## Experiment S2: Ultimatum Game as Proposer

The same participants who completed the TG-I in Experiment 1 also completed an ultimatum game in the role of proposer^2^. In the ultimatum game as proposer, participants were presented with a stake of 4 different sizes in a fixed order. For each trial, participants were asked to propose how the stake should be divided to an anonymous partner who they were informed would be another participant taking part in a similar experiment. Participants were also informed that the partner would be given the option to either accept or reject the proposal. Accepting the proposal meant that both participants would be paid according to the proposal whereas rejecting the proposal would mean that neither participant would receive anything.

We fit a mixed effects model with drug and gender predicting the proportion of the stake that was proposed to be given to the partner. The intercept was allowed to vary randomly for participants. There were no effects of either gender (*b* = -0.02 (0.03), *t* = -0.72, *p* = .47) or drug condition (*b* = 0.01 (0.03), *t* = 0.53, *p* = .60).

## Experiment S3: Ultimatum Game as Responder

In another experiment, participants (61 males, 57 females, 1 unknown; 61 acetaminophen, 58 placebo) completed an ultimatum game in the role of the responder (UG-R)^2^. The sample size was based on the effect size observed in another set of acetaminophen studies (Cohen’s *d* = .55)^3^. Participants participated in exchange for course credit. As all the other experiments, participants were financially compensated based on one randomly selected decision in addition to the course credit. Participants were unable to guess which drug they received (*χ*^2^ (1, *N* = 107) = 2.70, *p* = .10). Six participants were excluded from UG-R analyses for either not performing above chance on the task instructions quiz or expressing confusion about the UG-R to the experimenter during the session.

In the UG-R, participants received a pseudo-random series of 20 one-shot proposals from anonymous participants in Experiment S3. On each trial, a different partner proposed how to divide a stake (e.g., $10) with the responder (e.g., $6/$4). The stake amount was first displayed for 1500ms. Next, the proposed division was displayed and participants decided whether to accept or reject the offer. If they accepted, the stake was divided as proposed, but if they rejected then both players received nothing. The screens simultaneously presented the stakes and offers in both numerical and bar graph form (displays were modeled after those used by Crockett et al., 2008). The proposals presented to participants were selected so that they ranged from unfair (e.g., $9/$1) to fair (e.g., $5/$5). Besides being influenced by the fairness of the immediate proposal, acceptance decisions have also been shown to be affected by the fairness of previous proposals^5^. For example, a moderately fair proposal (e.g., $7/$3) is more likely to be accepted when it follows an unfair proposal (e.g., $9/$1) than when it follows a fair proposal (e.g., $5/$5). Therefore, we also tested whether acetaminophen influenced this effect. To calculate the change in offer fairness trial-to-trial, the proportion offered to the responder on the previous trial was subtracted from the proportion offered on the current trial.

First, we fit a full mixed effects logistic regression model with drug condition, proportion of stake offered to the participant, and their interaction, predicting offer acceptance. Offer fairness significantly influenced offer acceptance (*b* = 22.04 (2.43), *z* = 9.08, *p* < .001). There was no main effect of acetaminophen on proposal acceptance (*b* = -0.11 (0.57), *z* = -0.19, *p* = .85) and no interaction between drug condition and offer fairness, showing that it did not affect responder sensitivity to the fairness of the proposal (*b* = -0.57 (3.20), *z* = -0.18, *p* = .86; see Fig. S3 and Table S28).

To examine whether the influence of prior trials’ fairness on offer acceptance was affected by acetaminophen, we fit a second mixed effects logistic regression model with drug condition, change in fairness relative to prior trial, and their interaction. The model also controlled for the fairness of the immediate offer and its interaction with change in fairness. The intercept was allowed to vary randomly for participants. This model revealed a significant interaction between drug condition and the change in proposal fairness relative to the previous trial (i.e., proportion offered to the responder on the current trial minus the proportion offered on the previous trial) (*b* = -1.92 (0.88), *z* = -2.18, *p* = .029; see Fig. S6 and Table S29) such that the change in fairness had less of an influence among participants who received acetaminophen (*b* = 1.78 (0.68), *z* = 2.61, *p* = .009) relative to those who received placebo (*b* = 3.71 (0.77), *z* = 4.79, *p* < .001). Thus, because prior trials shape expectations for later trials^5^, we find evidence that acetaminophen reduced the relationship between expectations and behavior once again.

## Experiment S4: Trust Game as Trustee

The same participants who completed the UG-R in Experiment S3 also completed the trust game in the role of the trustee (TG-T). Specifically, participants were given a selection of 32 one-shot investments from anonymous partners and asked to decide how much of the investments they would return to the investors. Modeled off of a previous paradigm^6^, on each trial, trustees were first asked how much of the initial endowment they expected their partner (i.e. the investor) to invest. Next, trustees were informed of how much the investor actually chose to invest and this investment was multiplied times 4. Then, trustees were asked how much they thought the investor expected them to return (i.e., second-order beliefs). Finally, trustees were asked how much they would actually return and then were told how much the investor had actually expected. For all responses in this task, participants were presented with a scale from 0 to the maximum possible amount in 10% increments. During task instructions, participants were instructed that they would receive a bonus to their payment if their predictions about the investors’ expectations were accurate.

Past work has suggested that trustees will use their second-order beliefs when deciding how much to return in an effort to minimize potential guilt from violating their partners’ expectations. We hypothesized that acetaminophen might blunt these feelings of guilt (e.g., see Ref ^3^). Therefore, at the end of the experiment participants were presented with recaps of a subset of their decisions (i.e., 8 of the 32 trials) and asked how much guilt they would feel if they had returned a randomly selected alternative amount that was less than their actual return^6^. Participants rated the guilt they would experience if they had instead returned this hypothetical alternative amount on a 7-point scale (1 = Not at all; 7 = Extremely). One participant was excluded from TG-T analyses for not performing above chance on the task instructions quiz.

For each decision, the proportion of the initial endowment that was invested was calculated as well as the proportion of the amount the trustee received that the trustee believed the investor to expect to have returned. We fit a full mixed effects model with drug condition, second-order beliefs, and their interaction, predicting the amount returned. The model also controlled for the proportion of the initial endowment that was invested. One participant was identified as having a high degree of influence on the model fit and so was removed from the model (Cook’s D > 0.17; calculated with the influence.ME package^7^). There was no main effect of drug condition on the amount returned (*b* = -0.01 (0.02), *t* = -0.63, *p* = .53). However, there was a marginal interaction between drug condition and second-order beliefs on behavioral reciprocity (*b* = -0.10 (0.06), *t* = -1.79, *p* = .077; see Fig. S7 and Table S30) in the direction of second-order beliefs having a reduced relationship to actual reciprocity in the acetaminophen condition (*b* = 0.49 (0.04), *t* = 12.04, *p* < .001) relative to placebo (*b* = 0.59 (0.04), *t* = 14.69, *p* < .001). Thus, again we find evidence for a reduced relationship between a type of expectations (i.e., second-order beliefs) and behavior.

Next, we analyzed the effect of drug on counterfactual guilt. For each trial, the difference between the randomly generated alternative return amount and the actual amount returned was calculated. We fit a full mixed effects model with drug condition, difference between the counterfactual return (i.e., the alternative hypothetical return that was presented) and their actual return, and their interaction, predicting self-reported counterfactual guilt. Between- and within-participant variance were separated. Acetaminophen reduced counterfactual guilt as indicated by a marginal interaction between drug condition and counterfactual returns (*b* = 1.54 (0.90), *t* = 1.72, *p* = .089; see Fig. S8 and Table S31) such that the relationship between counterfactual returns and guilt was reduced on acetaminophen (*b* = -4.21 (0.69), *t* = -6.08, *p* < .001) relative to placebo (*b* = -5.74 (0.72), *t* = -8.01, *p* < .001).

## Experiment S5: Risk Game

The same participants who completed the TG-I in Experiment 2 also completed a risk game. In the risk game, participants were first given an endowment of points. They were then presented with a spinner that had been divided into four sections labelled: “0-1 points”, “1-2 points”, “2-3 points”, and “3-4 points”. Participants were instructed that could bet as many or as few of their endowment points as they wished. After placing their bet, the spinner would be spun and would stop on one of the four labelled sections. The points they bet would then be multiplied by a random number from within the range on the selected section and returned to them. For example, if a participant bet 100 points and the spinner stopped on “2-3 points”, the bet would be multiplied by a random number between 2 and 3 (e.g., 2.5) and the participant would receive the product (e.g., 250 points). After placing their bets, participants then were asked to report how points they expected to win. They were also asked to rate their anticipated affective response if they received the amount they expected to win using the same questions as in Experiment 2. Participants completed 4 trials, each with a different endowment size in random order: 60, 70, 90, 100. The sizes of the sections on the spinners were designed to match the probabilities of the returns that the investors in Experiment 1 received from their trustees. In this way, the risk game was designed to match the mechanics and probabilities of the TG-I except that there was no trustee and the winnings were determined randomly by the computer. This allowed us to examine whether acetaminophen influenced risk-taking behavior in a nonsocial context.

First, we calculated the proportion of the endowment that participants bet on each trial. We also calculated the proportion of the maximum possible win (i.e., maximum possible win was the bet times 4) on each trial that participants expected to win. We then fit a full mixed effects model with drug, self-reported expectations, and their interaction, predicting the proportion of the endowment that was bet. The intercept and slope for expectations were allowed to vary randomly for participants (see Table S32). Results revealed a main effect of participant gender on the amount of money that was bet such that women bet significantly less than men (*b* = -0.12 (0.04), *t* = -3.05, *p* = .003). However, there was no effect of drug condition (*b* = -0.05 (0.04), *t* = -1.40, *p* = .17) and no drug by expectations interaction (*b* = -0.19 (0.16), *t* = -1.18, *p* = .24). We also tested whether acetaminophen dampened anticipated affective responses to expected winnings but found no significant effects of drug condition (see Table S33). These results need to be replicated before drawing the conclusion that acetaminophen is specific for social risk. Furthermore, one methodological improvement would be to increase the variability in expectations between both participants and trials, which did not have the same variability as in the trust game.

# Tables

## Table S1. Descriptive Statistics

| **Variable** | | **Acetaminophen** | | **Placebo** | |
| --- | --- | --- | --- | --- | --- |
|  |  | ***M*** | ***SD*** | ***M*** | ***SD*** |
| Experiments 1-3 | Investment | 0.49 | 0.28 | 0.49 | 0.26 |
|  | Self-Reported Expected Return | 0.44 | 0.21 | 0.45 | 0.24 |
| Experiments 2-3 | Anticipated Affect | 0.03 | 0.72 | -0.07 | 0.87 |
| Experiments 4-5 | Investment | 0.42 | 0.24 | 0.37 | 0.22 |
|  | Self-Reported Expected Return | 0.50 | 0.21 | 0.50 | 0.20 |
|  | Anticipated Affect | -0.01 | 0.77 | -0.00 | 0.75 |
| Experiment S1 | Given | 0.43 | 0.22 | 0.42 | 0.19 |
| Experiment S2 | Given | 0.48 | 0.16 | 0.47 | 0.11 |
| Experiment S3 | Proposal Acceptance | 0.59 | 0.23 | 0.60 | 0.23 |
| Experiment S4 | Returned | 0.42 | 0.14 | 0.44 | 0.12 |
|  | Second-Order Belief | 0.50 | 0.04 | 0.50 | 0.08 |
|  | Counterfactual Guilt | 3.89 | 1.30 | 3.66 | 1.51 |
| Experiment S5 | Bet | 0.63 | 0.24 | 0.67 | 0.26 |
|  | Self-Reported Expected Win | 0.48 | 0.16 | 0.48 | 0.12 |
|  | Anticipated Affect | 0.03 | 0.81 | -0.04 | 0.70 |

## Table S2. Survey Data: Acetaminophen Usage Predicting Neighborhood Trust

|  | Model 1 | | | Model 2 | | | Model 3 | | |
| --- | --- | --- | --- | --- | --- | --- | --- | --- | --- |
|  | *ß* | SE | BCA 95% CI | *ß* | SE | BCA 95% CI | *ß* | SE | BCA 95% CI |
| Intercept | 0.00 | 0.02 | [-0.04, 0.03] | 0.01 | 0.03 | [-0.04, 0.06] | -0.02 | 0.03 | [-0.07, 0.04] |
| Acetaminophen Usage | -0.10 | 0.02 | [-0.14, -0.07] | -0.06 | 0.02 | [-0.10, -0.02] | -0.04 | 0.02 | [-0.08, -0.01] |
| Age |  |  |  | 0.14 | 0.02 | [0.11, 0.18] | 0.12 | 0.02 | [0.08, 0.16] |
| Sex |  |  |  | 0.17 | 0.03 | [0.11, 0.24] | 0.18 | 0.03 | [0.11, 0.25] |
| Household Income |  |  |  | 0.11 | 0.02 | [0.08, 0.15] | 0.10 | 0.02 | [0.06, 0.13] |
| Education |  |  |  | 0.05 | 0.02 | [0.01, 0.09] | 0.02 | 0.02 | [-0.02, 0.06] |
| Chronic Pain |  |  |  | -0.22 | 0.04 | [-0.29, -0.14] | -0.14 | 0.04 | [-0.22, -0.07] |
| Years in Current Residence |  |  |  |  |  |  | 0.07 | 0.02 | [0.04, 0.11] |
| Physical Health |  |  |  |  |  |  | 0.09 | 0.02 | [0.05, 0.14] |
| Mental/Emotional Health |  |  |  |  |  |  | 0.08 | 0.02 | [0.04, 0.13] |
| *Adj. R^2^* | .01 | | | .06 | | | .08 | | |

## Table S3. Survey Data: Acetaminophen Usage Predicting Social Integration

|  | Model 1 | | | Model 2 | | | Model 3 | | |
| --- | --- | --- | --- | --- | --- | --- | --- | --- | --- |
|  | *ß* | SE | BCA 95% CI | *ß* | SE | BCA 95% CI | *ß* | SE | BCA 95% CI |
| Intercept | -0.02 | 0.02 | [-0.05, 0.02] | 0.10 | 0.03 | [0.05, 0.16] | 0.08 | 0.03 | [0.03, 0.13] |
| Acetaminophen Usage | -0.07 | 0.02 | [-0.11, -0.04] | -0.06 | 0.02 | [-0.09, -0.02] | -0.04 | 0.02 | [-0.08, 0.00] |
| Age |  |  |  | 0.17 | 0.02 | [0.14, 0.21] | 0.13 | 0.02 | [0.09, 0.17] |
| Sex |  |  |  | -0.10 | 0.04 | [-0.17, -0.04] | -0.11 | 0.04 | [-0.17, -0.04] |
| Household Income |  |  |  | 0.08 | 0.02 | [0.04, 0.11] | 0.05 | 0.02 | [0.01, 0.09] |
| Education |  |  |  | 0.10 | 0.02 | [0.06, 0.14] | 0.07 | 0.02 | [0.03, 0.11] |
| Chronic Pain |  |  |  | -0.19 | 0.04 | [-0.27, -0.12] | -0.12 | 0.04 | [-0.19, -0.04] |
| Years in Current Residence |  |  |  |  |  |  | 0.10 | 0.02 | [0.06, 0.13] |
| Physical Health |  |  |  |  |  |  | 0.06 | 0.02 | [0.01, 0.10] |
| Mental/Emotional Health |  |  |  |  |  |  | 0.17 | 0.02 | [0.12, 0.21] |
| *Adj. R^2^* | .01 | | | .05 | | | .09 | | |

## Table S4. Survey Data: Ibuprofen Usage Predicting Neighborhood Trust

|  | Model 1 | | | Model 2 | | | Model 3 | | |
| --- | --- | --- | --- | --- | --- | --- | --- | --- | --- |
|  | *ß* | SE | BCA 95% CI | *ß* | SE | BCA 95% CI | *ß* | SE | BCA 95% CI |
| Intercept | 0.01 | 0.02 | [-0.02, 0.05] | 0.03 | 0.03 | [-0.02, 0.08] | 0.00 | 0.03 | [-0.05, 0.05] |
| Ibuprofen Usage | -0.03 | 0.02 | [-0.06, 0.01] | 0.02 | 0.02 | [-0.01, 0.06] | 0.02 | 0.02 | [-0.01, 0.06] |
| Age |  |  |  | 0.16 | 0.02 | [0.12, 0.2] | 0.13 | 0.02 | [0.1, 0.17] |
| Sex |  |  |  | 0.18 | 0.03 | [0.12, 0.25] | 0.19 | 0.03 | [0.12, 0.25] |
| Household Income |  |  |  | 0.11 | 0.02 | [0.08, 0.15] | 0.10 | 0.02 | [0.06, 0.14] |
| Education |  |  |  | 0.03 | 0.02 | [0, 0.07] | 0.01 | 0.02 | [-0.03, 0.05] |
| Chronic Pain |  |  |  | -0.24 | 0.04 | [-0.31, -0.17] | -0.16 | 0.04 | [-0.24, -0.09] |
| Years in Current Residence |  |  |  |  |  |  | 0.07 | 0.02 | [0.04, 0.11] |
| Physical Health |  |  |  |  |  |  | 0.09 | 0.02 | [0.05, 0.14] |
| Mental/Emotional Health |  |  |  |  |  |  | 0.07 | 0.02 | [0.03, 0.12] |
| *Adj. R^2^* | .001 | | | .06 | | | .08 | | |

## Table S5. Survey Data: Ibuprofen Usage Predicting Social Integration

|  | Model 1 | | | Model 2 | | | Model 3 | | |
| --- | --- | --- | --- | --- | --- | --- | --- | --- | --- |
|  | *ß* | SE | BCA 95% CI | *ß* | SE | BCA 95% CI | *ß* | SE | BCA 95% CI |
| Intercept | -0.02 | 0.02 | [-0.05, 0.02] | 0.11 | 0.03 | [0.06, 0.17] | 0.09 | 0.03 | [0.03, 0.14] |
| Ibuprofen Usage | -0.05 | 0.02 | [-0.08, -0.01] | -0.01 | 0.02 | [-0.05, 0.03] | -0.01 | 0.02 | [-0.05, 0.02] |
| Age |  |  |  | 0.18 | 0.02 | [0.14, 0.21] | 0.14 | 0.02 | [0.10, 0.18] |
| Sex |  |  |  | -0.12 | 0.04 | [-0.19, -0.05] | -0.13 | 0.03 | [-0.19, -0.06] |
| Household Income |  |  |  | 0.08 | 0.02 | [0.04, 0.11] | 0.06 | 0.02 | [0.02, 0.09] |
| Education |  |  |  | 0.10 | 0.02 | [0.06, 0.13] | 0.07 | 0.02 | [0.03, 0.11] |
| Chronic Pain |  |  |  | -0.17 | 0.04 | [-0.25, -0.10] | -0.09 | 0.04 | [-0.16, -0.01] |
| Years in Current Residence |  |  |  |  |  |  | 0.10 | 0.02 | [0.06, 0.13] |
| Physical Health |  |  |  |  |  |  | 0.07 | 0.02 | [0.02, 0.11] |
| Mental/Emotional Health |  |  |  |  |  |  | 0.16 | 0.02 | [0.11, 0.20] |
| *Adj. R^2^* | .002 | | | .04 | | | .09 | | |

## Table S6. Survey Data: Aspirin Usage Predicting Neighborhood Trust

|  | Model 1 | | | Model 2 | | | Model 3 | | |
| --- | --- | --- | --- | --- | --- | --- | --- | --- | --- |
|  | *ß* | SE | BCA 95% CI | *ß* | SE | BCA 95% CI | *ß* | SE | BCA 95% CI |
| Intercept | 0.00 | 0.02 | [-0.04, 0.04] | 0.05 | 0.03 | [-0.01, 0.11] | 0.02 | 0.03 | [-0.05, 0.08] |
| Aspirin Usage | -0.04 | 0.02 | [-0.08, 0.00] | -0.04 | 0.02 | [-0.08, 0.01] | -0.04 | 0.02 | [-0.08, 0.01] |
| Age |  |  |  | 0.12 | 0.02 | [0.08, 0.16] | 0.09 | 0.02 | [0.04, 0.14] |
| Sex |  |  |  | 0.13 | 0.04 | [0.04, 0.21] | 0.12 | 0.04 | [0.04, 0.20] |
| Household Income |  |  |  | 0.15 | 0.02 | [0.10, 0.19] | 0.13 | 0.02 | [0.08, 0.17] |
| Education |  |  |  | 0.02 | 0.02 | [-0.02, 0.07] | 0.00 | 0.02 | [-0.05, 0.04] |
| Chronic Pain |  |  |  | -0.26 | 0.04 | [-0.34, -0.17] | -0.17 | 0.05 | [-0.26, -0.08] |
| Years in Current Residence |  |  |  |  |  |  | 0.07 | 0.02 | [0.03, 0.12] |
| Physical Health |  |  |  |  |  |  | 0.08 | 0.03 | [0.02, 0.14] |
| Mental/Emotional Health |  |  |  |  |  |  | 0.10 | 0.03 | [0.04, 0.15] |
| *Adj. R^2^* | .001 | | | .05 | | | .08 | | |

## Table S7. Survey Data: Aspirin Usage Predicting Social Integration

|  | Model 1 | | | Model 2 | | | Model 3 | | |
| --- | --- | --- | --- | --- | --- | --- | --- | --- | --- |
|  | *ß* | SE | BCA 95% CI | *ß* | SE | BCA 95% CI | *ß* | SE | BCA 95% CI |
| Intercept | 0.00 | 0.02 | [-0.04, 0.04] | 0.13 | 0.03 | [0.07, 0.19] | 0.10 | 0.03 | [0.04, 0.16] |
| Aspirin Usage | -0.08 | 0.02 | [-0.12, -0.04] | -0.07 | 0.02 | [-0.12, -0.03] | -0.06 | 0.02 | [-0.10, -0.02] |
| Age |  |  |  | 0.19 | 0.02 | [0.15, 0.24] | 0.16 | 0.02 | [0.11, 0.20] |
| Sex |  |  |  | -0.13 | 0.04 | [-0.21, -0.05] | -0.14 | 0.04 | [-0.22, -0.06] |
| Household Income |  |  |  | 0.10 | 0.02 | [0.06, 0.15] | 0.08 | 0.02 | [0.04, 0.12] |
| Education |  |  |  | 0.09 | 0.02 | [0.05, 0.14] | 0.07 | 0.02 | [0.03, 0.12] |
| Chronic Pain |  |  |  | -0.22 | 0.04 | [-0.31, -0.13] | -0.12 | 0.05 | [-0.21, -0.03] |
| Years in Current Residence |  |  |  |  |  |  | 0.08 | 0.02 | [0.03, 0.12] |
| Physical Health |  |  |  |  |  |  | 0.05 | 0.03 | [0.00, 0.11] |
| Mental/Emotional Health |  |  |  |  |  |  | 0.19 | 0.03 | [0.14, 0.24] |
| *Adj. R^2^* | .006 | | | .07 | | | .11 | | |

## Table S8. Experiment 1: Drug X Expectations on Investment

Mixed effects model predicting amount invested. Expected Return was grand mean centered. Participants were treated as a random effect with varying intercepts (s^2^ = 0.05, SD = 0.22), varying slopes for Expected Return (s^2^ = 0.19, SD = 0.44), and their correlation (r = 0.05). N = 112.

|  | **Parameter Estimate** | **SE** | **t-Value** | **p-Value** |
| --- | --- | --- | --- | --- |
| Intercept | 0.47 | 0.03 | 13.54 | < .001 |
| Sex | -0.09 | 0.05 | -2.02 | .046 |
| Drug | 0.00 | 0.04 | 0.02 | .984 |
| Expected Return | 0.34 | 0.10 | 3.52 | < .001 |
| Drug * Expected Return | -0.30 | 0.14 | -2.17 | .034 |

## Table S9. Experiment 2: Drug X Expectations on Investment

Mixed effects model predicting amount invested. Expected Return was grand mean centered. Participants were treated as a random effect with varying intercepts (s^2^ = 0.05, SD = 0.23), varying slopes for Expected Return (s^2^ = 0.16, SD = 0.40), and their correlation (r = 0.07). N = 147.

|  | **Parameter Estimate** | **SE** | **t-Value** | **p-Value** |
| --- | --- | --- | --- | --- |
| Intercept | 0.57 | 0.04 | 14.74 | < .001 |
| Sex | -0.09 | 0.04 | -2.25 | .026 |
| Drug | -0.02 | 0.04 | -0.40 | .687 |
| Expected Return | 0.31 | 0.08 | 3.67 | < .001 |
| Drug * Expected Return | -0.26 | 0.11 | -2.29 | .025 |

## Table S10. Experiment 3: Drug X Expectations on Investment

Mixed effects model predicting amount invested. Expected Return was grand mean centered. Participants were treated as a random effect with varying intercepts (s^2^ = 0.07, SD = 0.26), varying slopes for Expected Return (s^2^ = 0.21, SD = 0.45), and their correlation (r = 0.16). N = 195.

|  | **Parameter Estimate** | **SE** | **t-Value** | **p-Value** |
| --- | --- | --- | --- | --- |
| Intercept | 0.56 | 0.04 | 15.85 | < .001 |
| Sex | -0.09 | 0.04 | -2.19 | .030 |
| Drug | 0.01 | 0.04 | 0.23 | .817 |
| Expected Return | 0.19 | 0.07 | 2.72 | .008 |
| Drug * Expected Return | -0.03 | 0.10 | -0.26 | .793 |

## Table S11. Experiments 1, 2, & 3 Combined: Drug X Expectations on Investment

Mixed effects model predicting amount invested. Expected Return was grand mean centered. Participants were treated as a random effect with varying intercepts (s^2^ = 0.06, SD = 0.24), varying slopes for Expected Return (s^2^ = 0.18, SD = 0.43), and their correlation (r = 0.10). N = 454.

|  | **Parameter Estimate** | **SE** | **t-Value** | **p-Value** |
| --- | --- | --- | --- | --- |
| Intercept | 0.54 | 0.02 | 25.57 | < .001 |
| Sex | -0.08 | 0.02 | -3.39 | < .001 |
| Drug | -0.00 | 0.02 | -0.17 | .866 |
| Expected Return | 0.26 | 0.05 | 5.65 | < .001 |
| Drug * Expected Return | -0.16 | 0.07 | -2.47 | .014 |

## Table S12. Experiments 1, 2, & 3 Combined: Drug on Expected Return

Mixed effects model predicting expected return. Participants were treated as a random effect with varying intercepts (s^2^ = 0.04, SD = 0.21). N = 454.

|  | **Parameter Estimate** | **SE** | **t-Value** | **p-Value** |
| --- | --- | --- | --- | --- |
| Intercept | 0.42 | 0.02 | 23.45 | < .001 |
| Sex | 0.06 | 0.02 | 2.65 | .008 |
| Drug | -0.01 | 0.02 | -0.29 | .774 |

## Table S13. Experiment 2: Effect of Drug on Anticipated Affective Responses to Expected Returns

Mixed effects model predicting affective response to expected return. Expected Return was grand mean centered. Participants were treated as a random effect with varying intercepts (s^2^ = 0.53, SD = 0.73), varying slopes for Expected Return (s^2^ = 2.67, SD = 1.63), and their correlation (r = -0.02). N = 154.

|  | **Parameter Estimate** | **SE** | **t-Value** | **p-Value** |
| --- | --- | --- | --- | --- |
| Intercept | -0.02 | 0.09 | -0.17 | .867 |
| Drug | 0.13 | 0.13 | 1.03 | .304 |
| Expected Return | 2.77 | 0.30 | 9.38 | < .001 |
| Drug * Expected Return | -0.70 | 0.40 | -1.74 | .085 |

## Table S14. Experiment 3: Effect of Drug on Anticipated Affective Responses to Expected Returns

Mixed effects model predicting affective response to expected return. Expected Return was grand mean centered. Participants were treated as a random effect with varying intercepts (s^2^ = 0.51, SD = 0.72), varying slopes for Expected Return (s^2^ = 4.29, SD = 2.07), and their correlation (r = 0.09). N = 197.

|  | **Parameter Estimate** | **SE** | **t-Value** | **p-Value** |
| --- | --- | --- | --- | --- |
| Intercept | 0.02 | 0.08 | 0.22 | .824 |
| Drug | 0.10 | 0.11 | 0.92 | .361 |
| Expected Return | 1.66 | 0.27 | 6.05 | < .001 |
| Drug * Expected Return | 0.82 | 0.39 | 2.12 | .036 |

## Table S15. Experiments 2 & 3 Combined: Effect of Drug on Anticipated Affective Responses to Expected Returns

Mixed effects model predicting affective response to expected return. Expected Return was grand mean centered. Participants were treated as a random effect with varying intercepts (s^2^ = 0.52, SD = 0.72), varying slopes for Expected Return (s^2^ = 3.75, SD = 1.94), and their correlation (r = 0.05). N = 351.

|  | **Parameter Estimate** | **SE** | **t-Value** | **p-Value** |
| --- | --- | --- | --- | --- |
| Intercept | 0.01 | 0.06 | 0.09 | .930 |
| Drug | 0.12 | 0.08 | 1.45 | .149 |
| Expected Return | 2.09 | 0.20 | 10.30 | < .001 |
| Drug * Expected Return | 0.21 | 0.28 | 0.73 | .464 |

## Table S16. Experiments 2 & 3 Combined: Effect of Drug on Anticipated Emotional Arousal to Expected Returns

Mixed effects model predicting emotional arousal response to expected return. Expected Return was grand mean centered. Participants were treated as a random effect with varying intercepts (s^2^ = 5.09, SD = 2.26), varying slopes for Expected Return (s^2^ = 14.45, SD = 3.80), and their correlation (r = 0.20). N = 351.

|  | **Parameter Estimate** | **SE** | **t-Value** | **p-Value** |
| --- | --- | --- | --- | --- |
| Intercept | 5.16 | 0.18 | 28.28 | < .001 |
| Drug | 0.45 | 0.25 | 1.79 | .075 |
| Expected Return | 3.12 | 0.45 | 6.91 | < .001 |
| Drug * Expected Return | -0.44 | 0.63 | -0.71 | .479 |

## Table S17. Experiments 2 & 3 Combined: Drug X Expectations X Weight (kg) on Investment

At the suggestion of an anonymous reviewer, both TG-I paradigms were analyzed as a function of self-reported body weight because the behavioral tests were started relatively early in the drug absorption phase^8^ when the effective concentration of drug in the brain is likely to be lower for those with a higher body weight.

In Experiments 2 and 3, participants were asked to self-report their weight. We did not have this data for participants in Experiment 1. This response was converted to kilograms. Then, in order to explore whether acetaminophen’s effect on the relationship between expected return and trust behavior was dose dependent, a model with a 3-way interaction between drug condition, expected return, and participant weight was fit to the data. If acetaminophen’s effect is dose dependent, low weight participants might exhibit a stronger effect than high weight participants if they received a greater milligram per kilogram dosage. Counter to this possibility, the 3-way interaction was nonsignificant (see Table S17). To illustrate the nonsignificant interaction, a median split based on participant weight was performed and then the original model was fit to high and low weight participants separately (see Tables S18 and S19).

Mixed effects model predicting amount invested. Both Expected Return and Weight were grand mean centered. Participants were treated as a random effect with varying intercepts (s^2^ = 0.06, SD = 0.25), varying slopes for Expected Return (s^2^ = 0.19, SD = 0.43), and their correlation (r = 0.12). N = 341.

|  | **Parameter Estimate** | **SE** | **t-Value** | **p-Value** |
| --- | --- | --- | --- | --- |
| Intercept | 0.56 | 0.03 | 20.46 | < .001 |
| Sex | -0.08 | 0.03 | -2.58 | .010 |
| Weight (kg) | 0.00 | 0.00 | 0.61 | .541 |
| Drug | -0.00 | 0.03 | -0.01 | .990 |
| Expected Return | 0.23 | 0.05 | 4.41 | < .001 |
| Weight (kg) * Drug | -0.00 | 0.00 | -0.13 | .893 |
| Weight (kg) * Expected Return | 0.00 | 0.00 | 0.57 | .570 |
| Drug * Expected Return | -0.13 | 0.07 | -1.74 | .083 |
| Weight (kg) * Drug * Expected Return | 0.00 | 0.00 | 0.85 | .396 |

## Table S18. Experiments 2 & 3 Combined: Drug X Expectations on Investment (Low Weight)

Mixed effects model predicting amount invested with low weight participants. Both Expected Return and Weight were grand mean centered. Participants were treated as a random effect with varying intercepts (s^2^ = 0.06, SD = 0.25), varying slopes for Expected Return (s^2^ = 0.27, SD = 0.52), and their correlation (r = 0.14). N = 171.

|  | **Parameter Estimate** | **SE** | **t-Value** | **p-Value** |
| --- | --- | --- | --- | --- |
| Intercept | 0.62 | 0.05 | 12.16 | < .001 |
| Sex | -0.17 | 0.05 | -3.38 | < .001 |
| Drug | -0.01 | 0.04 | -0.19 | .852 |
| Expected Return | 0.17 | 0.09 | 1.99 | .050 |
| Drug * Expected Return | -0.12 | 0.12 | -1.05 | .298 |

## Table S19. Experiments 2 & 3 Combined: Drug X Expectations on Investment (High Weight)

Mixed effects model predicting amount invested with high weight participants. Both Expected Return and Weight were grand mean centered. Participants were treated as a random effect with varying intercepts (s^2^ = 0.06, SD = 0.25), varying slopes for Expected Return (s^2^ = 0.10, SD = 0.32), and their correlation (r = 0.04). N = 170.

|  | **Parameter Estimate** | **SE** | **t-Value** | **p-Value** |
| --- | --- | --- | --- | --- |
| Intercept | 0.55 | 0.03 | 17.57 | < .001 |
| Sex | -0.02 | 0.04 | -0.51 | .610 |
| Drug | -0.00 | 0.04 | -0.11 | .915 |
| Expected Return | 0.29 | 0.06 | 4.68 | < .001 |
| Drug * Expected Return | -0.13 | 0.09 | -1.50 | .138 |

## Table S20. Experiment 4: Drug X Instructed Expectations on Investment

Mixed effects model predicting amount invested. Instructed Expected Return was grand mean centered. Participants were treated as a random effect with varying intercepts (s^2^ = 0.04, SD = 0.20), varying slopes for Instructed Expected Return (s^2^ = 0.21, SD = 0.46), and their correlation (r = 0.18). N = 266.

|  | **Parameter Estimate** | **SE** | **t-Value** | **p-Value** |
| --- | --- | --- | --- | --- |
| Intercept | 0.39 | 0.02 | 18.61 | < .001 |
| Sex | -0.09 | 0.02 | -3.60 | < .001 |
| Drug | 0.05 | 0.02 | 1.94 | .053 |
| Expected Return | 0.75 | 0.04 | 18.18 | < .001 |
| Drug * Expected Return | -0.01 | 0.06 | -0.17 | .868 |

## Table S21. Experiment 5: Drug X Instructed Expectations on Investment

Mixed effects model predicting amount invested. Instructed Expected Return was grand mean centered. Participants were treated as a random effect with varying intercepts (s^2^ = 0.07, SD = 0.26), varying slopes for Instructed Expected Return (s^2^ = 0.31, SD = 0.55), and their correlation (r = 0.09). N = 202.

|  | **Parameter Estimate** | **SE** | **t-Value** | **p-Value** |
| --- | --- | --- | --- | --- |
| Intercept | 0.45 | 0.03 | 14.29 | < .001 |
| Sex | -0.09 | 0.04 | -2.37 | .019 |
| Drug | 0.03 | 0.04 | 0.81 | .422 |
| Expected Return | 0.77 | 0.06 | 13.47 | < .001 |
| Drug * Expected Return | -0.05 | 0.08 | -0.61 | .543 |

## Table S22. Experiments 4 & 5 Combined: Drug X Instructed Expectations on Investment

Mixed effects model predicting amount invested. Instructed Expected Return was grand mean centered. Participants were treated as a random effect with varying intercepts (s^2^ = 0.05, SD = 0.23), varying slopes for Instructed Expected Return (s^2^ = 0.27, SD = 0.52), and their correlation (r = 0.14). N = 468.

|  | **Parameter Estimate** | **SE** | **t-Value** | **p-Value** |
| --- | --- | --- | --- | --- |
| Intercept | 0.42 | 0.02 | 23.12 | < .001 |
| Sex | -0.09 | 0.02 | -4.07 | < .001 |
| Drug | 0.04 | 0.02 | 1.84 | .067 |
| Expected Return | 0.76 | 0.03 | 22.32 | < .001 |
| Drug * Expected Return | -0.03 | 0.05 | -0.56 | .576 |

## Table S23. Experiments 4 & 5 Combined: Drug X Self-Reported Expectations on Investment

Mixed effects model predicting amount invested. Self-Reported Expected Return was grand mean centered. Participants were treated as a random effect with varying intercepts (s^2^ = 0.14, SD = 0.37), varying slopes for Self-Reported Expected Return (s^2^ = 2.56, SD = 1.60), and their correlation (r = 0.48). N = 460.

|  | **Parameter Estimate** | **SE** | **t-Value** | **p-Value** |
| --- | --- | --- | --- | --- |
| Intercept | 0.49 | 0.03 | 16.33 | < .001 |
| Sex | -0.12 | 0.03 | -3.88 | < .001 |
| Drug | 0.03 | 0.04 | 0.93 | .351 |
| Expected Return | 0.45 | 0.12 | 3.83 | < .001 |
| Drug * Expected Return | 0.04 | 0.16 | 0.26 | .792 |

## Table S24. Experiments 4 & 5 Combined: Effect of Drug on Anticipated Affective Responses to Self-Reported Expected Returns

Mixed effects model predicting anticipated affective response to self-reported expected return. Self-Reported Expected Return was grand mean centered. Participants were treated as a random effect with varying intercepts (s^2^ = 1.05, SD = 1.03), varying slopes for Self-Reported Expected Return (s^2^ = 13.15, SD = 3.63), and their correlation (r = 0.24). N = 462.

|  | **Parameter Estimate** | **SE** | **t-Value** | **p-Value** |
| --- | --- | --- | --- | --- |
| Intercept | 0.12 | 0.07 | 1.64 | .102 |
| Drug | -0.01 | 0.10 | -0.14 | .892 |
| Expected Return | 3.09 | 0.27 | 11.28 | < .001 |
| Drug * Expected Return | -0.24 | 0.38 | -0.62 | .535 |

## Table S25. Experiments 4 & 5 Combined: Drug X Instructed Expectations X Weight on Investment

At the suggestion of an anonymous reviewer, both TG-I paradigms were analyzed as a function of self-reported body weight because the behavioral tests were started relatively early in the drug absorption phase^8^ when the effective concentration of drug in the brain is likely to be lower for those with a higher body weight.

In Experiments 4 and 5, participants were asked to self-report their weight. This response was converted to kilograms. Then, in order to explore whether acetaminophen’s effect was dose dependent, a model with an interaction between drug condition and participant weight was fit to the data. Initially the full 3-way interaction between drug condition, expected return, and participant weight was included, but the terms for the 3-way interaction as well as the 2-way interaction between participant weight and expected return were dropped from the final model because neither effect was significant nor theoretically relevant. If acetaminophen’s effect is dose dependent, low weight participants should exhibit a stronger effect that high weight participants because they received a greater milligram per kilogram dosage. There was not a significant interaction between drug condition and participant weight (see Table S25). Nevertheless, to explore further, a median split based on participant weight was performed and then the original model was fit to high and low weight participants separately (see Tables S26 and S27). This analysis did reveal that low weight participants showed a significant main effect of drug condition whereas high weight participants did not (see Figure S5), which is suggestive of a weak dose-dependent effect.

Mixed effects model predicting amount invested. Both Instructed Expected Return and Weight were grand mean centered. Participants were treated as a random effect with varying intercepts (s^2^ = 0.05, SD = 0.23), varying slopes for Instructed Expected Return (s^2^ = 0.27, SD = 0.52), and their correlation (r = 0.14). N = 468.

|  | **Parameter Estimate** | **SE** | **t-Value** | **p-Value** |
| --- | --- | --- | --- | --- |
| Intercept | 0.42 | 0.02 | 22.28 | < .001 |
| Sex | -0.08 | 0.02 | -3.52 | < .001 |
| Weight (kg) | 0.00 | 0.00 | 0.67 | .502 |
| Drug | 0.04 | 0.02 | 1.85 | .065 |
| Expected Return | 0.76 | 0.03 | 22.32 | < .001 |
| Weight (kg) * Drug | -0.00 | 0.00 | -0.96 | .336 |
| Drug * Expected Return | -0.03 | 0.05 | -0.56 | .576 |

## Table S26. Experiments 4 & 5 Combined: Drug X Instructed Expectations on Investment (Low Weight)

Mixed effects model predicting amount invested with low weight participants. Both Instructed Expected Return and Weight were grand mean centered. Participants were treated as a random effect with varying intercepts (s^2^ = 0.05, SD = 0.23), varying slopes for Instructed Expected Return (s^2^ = 0.24, SD = 0.49), and their correlation (r = 0.13). N = 238.

|  | **Parameter Estimate** | **SE** | **t-Value** | **p-Value** |
| --- | --- | --- | --- | --- |
| Intercept | 0.42 | 0.03 | 13.48 | < .001 |
| Sex | -0.09 | 0.03 | -2.86 | .005 |
| Drug | 0.06 | 0.03 | 2.17 | .031 |
| Expected Return | 0.77 | 0.04 | 17.65 | < .001 |
| Drug * Expected Return | -0.09 | 0.06 | -1.40 | .162 |

## Table S27. Experiments 4 & 5 Combined: Drug X Instructed Expectations on Investment (High Weight)

Mixed effects model predicting amount invested with high weight participants. Both Instructed Expected Return and Weight were grand mean centered. Participants were treated as a random effect with varying intercepts (s^2^ = 0.05, SD = 0.23), varying slopes for Instructed Expected Return (s^2^ = 0.30, SD = 0.55), and their correlation (r = 0.15). N = 230.

|  | **Parameter Estimate** | **SE** | **t-Value** | **p-Value** |
| --- | --- | --- | --- | --- |
| Intercept | 0.43 | 0.02 | 18.24 | < .001 |
| Sex | -0.09 | 0.04 | -2.54 | .012 |
| Drug | 0.01 | 0.03 | 0.47 | .640 |
| Expected Return | 0.75 | 0.05 | 14.13 | < .001 |
| Drug * Expected Return | 0.03 | 0.07 | 0.44 | .660 |

## Table S28. Experiment S3: Drug X Proposal Fairness on Proposal Acceptance

Mixed effects logistic regression predicting proposal acceptance. Participants were treated as a random effect with varying intercepts (s^2^ = 7.82, SD = 2.80), varying slopes for proposal fairness (s^2^ = 175.67, SD = 13.25), and their correlation (r = -0.76). N = 113.

|  | **Parameter Estimate** | **SE** | **z-Value** | **p-Value** |
| --- | --- | --- | --- | --- |
| Intercept | 0.69 | 0.42 | 1.66 | .096 |
| Drug | -0.11 | 0.57 | -0.19 | .853 |
| Proposal Fairness | 22.04 | 2.43 | 9.06 | < .001 |
| Drug * Proposal Fairness | -0.57 | 3.21 | -0.18 | .859 |

## Table S29. Experiment S3: Drug X Change in Proposal Fairness on Proposal Acceptance

Mixed effects logistic regression predicting proposal acceptance. Participants were treated as a random effect with varying intercepts (s^2^ = 7.35, SD = 2.71). Random slopes were not included in this model because they prevented model convergence. N = 113.

|  | **Parameter Estimate** | **SE** | **z-Value** | **p-Value** |
| --- | --- | --- | --- | --- |
| Intercept | 0.63 | 0.40 | 1.58 | .114 |
| Proposal Fairness | 17.20 | 1.23 | 14.02 | < .001 |
| Change in Fairness | 3.71 | 0.77 | 4.79 | < .001 |
| Drug | -0.11 | 0.54 | -0.20 | .845 |
| Proposal Fairness * Change in Fairness | 21.57 | 3.90 | 5.53 | < .001 |
| Change in Fairness * Drug | -1.92 | 0.88 | -2.18 | .029 |

## Table S30. Experiment S4: Drug X Second-Order Beliefs on Amount Returned

Mixed effects model predicting amount returned. Amount Invested and Second-Order Beliefs were both grand mean centered. Participants were treated as a random effect with varying intercepts (s^2^ = 0.01, SD = 0.12), varying slopes for Amount Invested (s^2^ = 0.01, SD = 0.12), varying slopes for Second-Order Beliefs (s^2^ = 0.06, SD = 0.24), and their correlations (intercept and Amount Invested: r = 0.08; intercept and Second-Order Beliefs: r = 0.57; Amount Invested and Second-Order Beliefs: r = -0.26). N = 117.

|  | **Parameter Estimate** | **SE** | **t-Value** | **p-Value** |
| --- | --- | --- | --- | --- |
| Intercept | 0.44 | 0.02 | 27.55 | < .001 |
| Proportion Invested | 0.08 | 0.01 | 5.42 | < .001 |
| Drug | -0.01 | 0.02 | -0.63 | .531 |
| Second-Order Belief | 0.59 | 0.04 | 14.69 | < .001 |
| Drug * Second-Order Belief | -0.10 | 0.06 | -1.79 | .077 |

## Table S31. Experiment S4: Drug X Counterfactual Return on Counterfactual Guilt

Mixed effects model predicting self-reported counterfactual guilt. The difference between the counterfactual return (i.e., the alternative hypothetical return that was presented) and their actual return was calculated (here labeled simply “counterfactual return”). Counterfactual Return and the Actual Amount Returned were grand mean centered. Participants were treated as a random effect with varying intercepts (s^2^ = 1.54, SD = 1.24), varying slopes for Actual Return (s^2^ = 10.86, SD = 3.30), varying slopes for Counterfactual Return (s^2^ = 12.51, SD = 3.54), and their correlations (intercept and Actual Return: r = -0.23; intercept and Counterfactual Return: r = -0.54; Actual Return and Counterfactual Return: r = 0.50). N = 118.

|  | **Parameter Estimate** | **SE** | **t-Value** | **p-Value** |
| --- | --- | --- | --- | --- |
| Intercept | 3.79 | 0.17 | 21.68 | < .001 |
| Actual Return | -3.07 | 0.49 | -6.26 | < .001 |
| Counterfactual Return | -5.74 | 0.72 | -8.01 | < .001 |
| Drug | 0.34 | 0.24 | 1.39 | .166 |
| Actual Return * Counterfactual Return | 7.43 | 1.43 | 5.18 | < .001 |
| Counterfactual Return * Drug | 1.54 | 0.90 | 1.72 | .089 |

## Table S32. Experiment S5: Drug X Expectations on Bet

Mixed effects model predicting amount bet. Expected Win was grand mean centered. Participants were treated as a random effect with varying intercepts (s^2^ = 0.05, SD = 0.22), varying slopes for Expected Win (s^2^ = 0.21, SD = 0.45), and their correlation (r = 0.07). N = 151.

|  | **Parameter Estimate** | **SE** | **t-Value** | **p-Value** |
| --- | --- | --- | --- | --- |
| Intercept | 0.76 | 0.04 | 20.81 | < .001 |
| Sex | -0.12 | 0.04 | -3.05 | .003 |
| Drug | -0.05 | 0.04 | -1.40 | .165 |
| Expected Win | 0.18 | 0.12 | 1.49 | .140 |
| Drug * Expected Win | -0.19 | 0.16 | -1.18 | .243 |

## Table S33. Experiment S5: Drug X Expectations on Anticipated Affective Responses to Expected Win

Mixed effects model predicting affective response to expected win. Expected Win was grand mean centered. Participants were treated as a random effect with varying intercepts (s^2^ = 0.36, SD = 0.60), varying slopes for Expected Win (s^2^ = 2.96, SD = 1.72), and their correlation (r = -0.11). N = 158.

|  | **Parameter Estimate** | **SE** | **t-Value** | **p-Value** |
| --- | --- | --- | --- | --- |
| Intercept | -0.02 | 0.08 | -0.26 | .796 |
| Drug | 0.06 | 0.11 | 0.58 | .562 |
| Expected Win | 2.61 | 0.40 | 6.50 | < .001 |
| Drug * Expected Win | -0.65 | 0.55 | -1.17 | .243 |

# Task Instructions and Quizzes

## Experiments 1, S1, & S2: General Instructions

Please read over the following instructions regarding the next set of decision-making tasks. If you have any questions, read back through these instructions. If you still have questions after reading through the instructions again, please get the experimenter before going any further.

For each decision in the decision-making tasks that follow, you will be randomly paired with different people who are completing a similar experiment at different days and times. You will not be told who these people are either during or after the experiment and these people will not be told who you are. You will notice that there are other people in your current session. You will not be paired with any of these people.

For each task, you will be given a number of points and asked to make a decision. Please read the instructions for each task carefully because the exact setup for each decision-making task will vary.

Treat the points in this task as you would real money because once the experiment has finished we will convert these points to real money and you will receive a payment corresponding to one of your decisions. The decision used to determine your payment will be randomly selected.

For this experiment, points will be converted to money at a rate of 2.5 cents for every point. Thus, 200 points equals $5.00.

## Experiment S1: Dictator Game Instructions

Please read over the following instructions regarding the first decision-making task. If you have any questions, read back through these instructions. If you still have questions after reading through the instructions again, please get the experimenter before going any further.

In this task, you will be paired with a different person who is completing a similar experiment at a different day and time. You will not be told who this person is either during or after the experiment and this person will not be told who you are. You will notice that there are other people in your current session. You will not be paired with any of these people.

For each round of this task, you will be asked to propose how a set number of points will be divided between yourself and your partner. You may choose to send some, all, or none of the points to your partner. Your final points for each round will equal the number of points you assign to yourself and your partner will receive the number of points you assign to him/her.

Treat the points in this task as you would real money because once the experiment has finished we will convert these points to real money and you will receive a payment corresponding to one of your decisions. The decision used to determine your payment will be randomly selected.

For this experiment, points will be converted to money at a rate of 2.5 cents for every point. Thus, 200 points equals $5.00.

## Experiment 1: Trust Game as Investor Instructions

Please read over the following instructions regarding the second decision-making task. If you have any questions, read back through these instructions. If you still have questions after reading through the instructions again, please get the experimenter before going any further.

In this task, you will be paired with a different person who is completing a similar experiment at a different day and time. You will not be told who this person is either during or after the experiment and this person will not be told who you are. You will notice that there are other people in your current session. You will not be paired with any of these people.

For each round of this task, you will be given a number of points. You will then be asked how many of your points you would like to send to your partner. You may choose to send some, all, or none of your initial point endowment. Any points you send to your partner will be multiplied by 4. For example, if you send 5 points your partner will receive 20 points. Your partner will be asked to decide how many of these points to send back to you and how many to keep. Your final points for each round will equal the number of points you kept for yourself plus the points your partner sends back to you.

Treat the points in this task as you would real money because once the experiment has finished we will convert these points to real money and you will receive a payment corresponding to one of your decisions. The decision used to determine your payment will be randomly selected.

For this experiment, points will be converted to money at a rate of 2.5 cents for every point. Thus, 200 points equals $5.00.

## Experiment S2: Ultimatum Game as Proposer Instructions

Please read over the following instructions regarding the third decision-making task. If you have any questions, read back through these instructions. If you still have questions after reading through the instructions again, please get the experimenter before going any further.

In this task, you will be paired with a different person who is completing a similar experiment at a different day and time. You will not be told who this person is either during or after the experiment and this person will not be told who you are. You will notice that there are other people in your current session. You will not be paired with any of these people.

For each round of this task, you will be asked to propose how a set number of points will be divided between yourself and your partner. You may choose to send some, all, or none of the points to your partner. Your partner will receive your proposal for how to divide the points and will be given a chance to accept or reject the offer. If your partner accepts the proposal, then the points will be divided as you have specified in your proposal. If your partner rejects the proposal, then both of you will receive zero points.

Treat the points in this task as you would real money because once the experiment has finished we will convert these points to real money and you will receive a payment corresponding to one of your decisions. The decision used to determine your payment will be randomly selected.

For this experiment, points will be converted to money at a rate of 2.5 cents for every point. Thus, 200 points equals $5.00.

## Experiment 2: General Instructions

Please read over the following instructions regarding the next set of decision-making tasks. If you have any questions, read back through these instructions. If you still have questions after reading through the instructions again, please get the experimenter before going any further.

For each decision in the decision-making tasks that follow, you will be randomly paired with different people who are completing a similar experiment at different days and times. You will not be told who these people are either during or after the experiment and these people will not be told who you are. You will notice that there are other people in your current session. You will not be paired with any of these people.

For each task, you will be given a number of points and asked to make a decision. Please read the instructions for each task carefully because the exact setup for each decision-making task will vary.

Treat the points in this task as you would real money because once the experiment has finished we will convert these points to real money and you will receive a payment corresponding to one of your decisions. The decision used to determine your payment will be randomly selected.

For this experiment, points will be converted to money at a rate of 2.5 cents for every point. Thus, 200 points equals $5.00.

## Experiment 2: General Instructions Quiz

What determines how many points you end up earning in each round?

- The points are randomly generated by the computer
- **My decisions and the decisions of participants in other related experiments.**
- My decisions and the decisions of other participants in this same experimental session.

Will you receive actual money for completing these tasks?

- No, the points and money are imaginary
- **Yes, the points and money are real and everyone will receive a payment for one of their decisions.**
- Yes, but not everyone will actually get paid.

Will your partners ever find out who you are?

- Yes, my name will be given to them along with the results of my decision.
- They won't know my name, but will be given other information about me.
- **No, all my decisions will be completely anonymous.**

## Experiment 2: Trust Game as Investor Instructions

Please read over the following instructions regarding the next decision-making task. If you have any questions, read back through these instructions. If you still have questions after reading through the instructions again, please get the experimenter before going any further.

In this task, you will be paired with a different person who is completing a similar experiment at a different day and time. You will not be told who this person is either during or after the experiment and this person will not be told who you are. You will notice that there are other people in your current session. You will not be paired with any of these people.

For each round of this task, you will be given a number of points. You will then be asked how many of your points you would like to send to your partner. You may choose to send some, all, or none of your initial point endowment. Any points you send to your partner will be multiplied by 4. For example, if you send 5 points your partner will receive 20 points. Your partner will be asked to decide how many of these points to send back to you and how many to keep. Your final points for each round will equal the number of points you kept for yourself plus the points your partner sends back to you.

Treat the points in this task as you would real money because once the experiment has finished we will convert these points to real money and you will receive a payment corresponding to one of your decisions. The decision used to determine your payment will be randomly selected.

For this experiment, points will be converted to money at a rate of 2.5 cents for every point. Thus, 200 points equals $5.00.

## Experiment 2: Trust Game as Investor Quiz

If you decided to send 50 points to your partner, how many points would your partner receive from you?

- 50
- 100
- **200**

What determines how many points will get returned to you?

- A computer randomly generates an amount.
- **A participant in another experimental session who will act as your partner.**
- A participant in the same experimental session as you who will act as your partner.

## Experiment 3: Instructions

Please read over the following instructions regarding the next decision-making task. If you have any questions, please get the experimenter before going any further.

In this task, you will be paired with a different person who is completing a similar experiment at a different day and time. You will not be told who this person is either during or after the experiment and this person will not be told who you are. They will not receive any other information about you either.

For each round of this task, you will be given an amount of points. You will then choose how many of your points you would like to send to your partner. You may choose to send some, all, or none of your points.

Any points you send to your partner will be multiplied by 4. For example, if you send 5 points your partner will receive 20 points. Your partner will be asked to decide how many of these points to send back to you and how many to keep. NOTE: the numbers used here are only examples.

The total number of points you earn will be the number of points you kept for yourself plus the points your partner sends back to you.

Treat the points in this task as you would real money because once the experiment has finished we will convert these points to real money and both you and your partner will receive payments corresponding to your decisions. The decision used to determine your payment will be randomly selected.

For this experiment, points will be converted to money at a rate of 2.5 cents for every point. Thus, 200 points equals $5.00.

## Experiment 3: Instructions Quiz

If you decided to send 50 points to your partner, how many points would your partner receive from you?

- 50
- 100
- **200**

What determines how many points will get returned to you?

- A computer randomly generates an amount.
- **A participant in another experimental session who you have been paired with as your partner.**
- A computer randomly selects an amount to return from the set of return amounts equal to the set of amounts all possible partners return.

Who will receive money for completing these tasks?

- No one will be paid. The points and money are imaginary.
- **Both my partner and I will be paid. The points and money are real and everyone will receive a payment for one of their decisions.**
- I will be paid but my partner will not. The points and money are real and I will receive a payment for one of my decisions, but my partner will not receive any money.

Will your partners ever find out who you are?

- Yes, my name will be given to them along with the results of my decision.
- They won't know my name, but will be given other information about me.
- **No, all my decisions will be completely anonymous.**

## Experiment 4: General Instructions

Please read over the following instructions regarding the next set of decision-making tasks. If you have any questions, read back through these instructions. If you still have questions after reading through the instructions again, please get the experimenter before going any further.

For each of the decision-making tasks that follow, you will be randomly paired with different people who are completing a similar experiment at different days and times. You will not be told who these people are during or after the experiment. These people will not be told who you are. You will notice that there are other people in your current session. You will not be paired with any of these people.

For each task, you will be given a number of points and asked to make a decision. Please read the instructions for each task carefully because the exact setup for each decision-making task will vary.

Once the experiment has finished we will convert the points from one of your decisions to real money, which you will receive. The decision used to determine your payment will be randomly selected. Therefore, you should treat each decision as if it’s the one that counts for real money.

For this experiment, points will be converted to money at a rate of 2.5 cents for every point. Thus, 200 points equals $5.00.

## Experiment 4: General Instructions Quiz

What determines how many points you end up earning in each round?

- The points are randomly generated by the computer.
- **My decision and the decision of a participant in another experimental session.**
- My decision and the decision of a participant in this same experimental session.

Who will receive money for completing these tasks?

- No one will be paid. The points and money are imaginary.
- **Both my partner and I will be paid. The points and money are real and everyone will receive a payment for one of their decisions.**
- I will but my partner will not. The points and money are real and I will receive a payment for one of my decisions, but my partner will not receive any money.

Will your partners ever find out who you are?

- Yes, my name will be given to them along with the results of my decision.
- They won't know my name, but will be given other information about me.
- **No, all my decisions will be completely anonymous.**

## Experiment 4: Trust Game as Investor Instructions

For each round of this task, you will be given a number of points. You will then be asked how many of your points you would like to send to your partner. You may choose to send some, all, or none of your initial point endowment. Any points you send to your partner will be multiplied by 4. For example, if you send 5 points your partner will receive 20 points. Your partner will be asked to decide how many of these points to send back to you and how many to keep. The total number of points you earn will be the number of points you kept for yourself plus the points your partner sends back to you.

Each of your partners have already completed this task and so we will be able to tell you what percentage of the points they return ON AVERAGE.

Once the experiment has finished we will convert the points from one of your decisions to real money, which you will receive. The decision used to determine your payment will be randomly selected. Therefore, you should treat each decision as if it’s the one that counts for real money.

For this experiment, points will be converted to money at a rate of 2.5 cents for every point. Thus, 200 points equals $5.00.

## Experiment 4: Trust Game as Investor Quiz

If you decided to send 50 points to your partner, how many points would your partner receive from you?

- 50
- 100
- **200**

What determines how many points get returned to you?

- A computer randomly chooses.
- **A participant in another experimental session who you are partnered with.**
- A participant in the same experimental session who you are partnered with.

## Experiment 5: Instructions

Please read over the following instructions regarding the next decision-making task. If you have any questions, please get the experimenter before going any further.

The next task will be the same as the task you just completed.

However, each of your partners have completed this task previously with different partners. We will tell you what percentage of the points they received that they returned to these partners ON AVERAGE. NOTE: these percentages tell you what each partner has done in the past, not what they have decided for you.

The total number of points you earn will be the number of points you kept for yourself plus the points your partner sends back to you.

Treat the points in this task as you would real money because once the experiment has finished we will convert these points to real money and both you and your partner will receive payments corresponding to your decisions. The decision used to determine your payment will be randomly selected.

For this experiment, points will be converted to money at a rate of 2.5 cents for every point. Thus, 200 points equals $5.00.

## Experiment 5: Instructions Quiz

If you decided to send 25 points to your partner, how many points would your partner receive from you?

- 50
- **100**
- 200

What determines how many points will get returned to you?

- A computer randomly generates an amount.
- **A participant in another experimental session who you have been paired with as your partner.**
- A computer randomly selects an amount to return from the set of return amounts equal to the set of amounts all possible partners return.

Who will receive money for completing these tasks?

- No one will be paid. The points and money are imaginary.
- **Both my partner and I will be paid. The points and money are real and everyone will receive a payment for one of their decisions.**
- I will be paid but my partner will not. The points and money are real and I will receive a payment for one of my decisions, but my partner will not receive any money.

Will your partners ever find out who you are?

- Yes, my name will be given to them along with the results of my decision.
- They won't know my name, but will be given other information about me.
- **No, all my decisions will be completely anonymous.**

On each round, what additional piece of information will you be told about each partner?

- The percentage of points that they will return.
- **The percentage of points they have returned ON AVERAGE in this task in the past with other partners.**
- The percentage of points that I should send to them.

## Experiments S3 & S4: General Instructions

Please read over the following instructions regarding the next set of decision-making tasks. If you have any questions, read back through these instructions. If you still have questions after reading through the instructions again, please get the experimenter before going any further.

For each decision in the decision-making tasks that follow, you will be randomly paired with different people who completed their part of the tasks earlier this semester. You will not be told who these people are either during or after the experiment and these people will not be told who you are. You will notice that there are other people in your current session. You will not be paired with any of these people.

For each task, your partners were given a number of points and asked to make a decision. You will be receiving points based on their decisions and will be asked to make decisions yourself about the points you receive. Please read the instructions for each task carefully because the exact setup for each decision-making task will vary.

Treat the points in these tasks as you would real money because once the experiment has finished we will convert these points to real money and you will receive a payment corresponding to one of your decisions. The decision used to determine your payment will be randomly selected.

For this experiment, points will be converted to money at a rate of 2.5 cents for every point. Thus, 200 points equals $5.00.

## Experiments S3 & S4: General Instructions Quiz

What determines how many points you receive from your partner each round?

- The points are randomly generated by the computer.
- **The points are sent from participants who completed an experiment earlier this semester.**
- The points are being sent from another participant in this same session.

Will you and your partners receive actual money for completing these tasks?

- No, the points and money are imaginary.
- I will but my partners will not.
- **Yes, the points and money are real and we will both receive payment for one of our decisions.**

Will your partners ever find out who you are?

- Yes, my name will be given to them along with the results of my decision.
- They won't know my name, but will be given other information about me.
- **No, all my decisions will be completely anonymous.**

##

## Experiment S3: Ultimatum Game Instructions

Please read over the following instructions regarding the second decision-making task. If you have any questions, read back through these instructions. If you still have questions after reading through the instructions again, please get the experimenter before going any further.

In this task, your partners were each given a number of points. They were then asked to propose how to divide the points between themselves and you. They were told that you would be asked to asked to decide whether to accept or reject the proposal.

For each round of this task, you will receive a proposal from a different partner. Your task is to decide whether you would like to accept or reject the proposal. If you accept the proposal, then the points will be divided as your partner has proposed. If you reject the proposal, then both of you will receive zero points.

Treat the points in this task as you would real money because once the experiment has finished we will convert these points to real money and you will receive a payment corresponding to one of your decisions. The decision used to determine your payment will be randomly selected.

## Experiment S3: Ultimatum Game Quiz

Each round of this game is played with:

- The computer
- The same partner each time
- **A different partner each time**

What happens if you accept a proposal?

- Neither of you get any points
- You will be given the opportunity to make your own proposal in return
- **You and your partner will receive the points specified in the proposal**

What happens if you reject a proposal?

- **Neither of you get any points**
- You will be given the opportunity to make your own proposal in return
- You and your partner will receive the points specified in the proposal

## Experiment S4: Trust Game as Trustee Instructions

Please read over the following instructions regarding the first decision-making task. If you have any questions, read back through these instructions. If you still have questions after reading through the instructions again, please get the experimenter before going any further.

In this task, your partners were each given a number of points. They were then asked how many of their points they would like to send to you. Any points they sent to you would be multiplied by 4. For example, if your partner sent 5 points you will receive 20 points. They were told that you would be asked to decide how many of these points to send back to them and how many to keep for yourself. They were also asked how many points they expected that you would send back to them.

For each round of this task, you will receive points from a different partner. Your task is decide how many points to send back to your partner. You may choose to send back some, all, or none of the points you receive. Your final points for each round will equal the number of points you keep for yourself. Your partners' final points will equal the number of points you send back to them plus the points they kept for themselves initially.

During this task, you will also be asked to make an estimate about how many points you think a partner expects you to send back. Please try to be accurate in your estimates. If you achieve an acceptable level of accuracy, you will receive a bonus to your final payment.

Treat the points in this task as you would real money because once the experiment has finished we will convert these points to real money and you will receive a payment corresponding to one of your decisions. The decision used to determine your payment will be randomly selected.

## Experiment S4: Trust Game as Trustee Quiz

Each round of this game is played with:

- The computer
- The same partner each time
- **A different partner each time**

What happens to the points your partner chose to send to you?

- **They are multiplied by 4**
- They are multiplied by 2
- They remain the same

What happens if you are accurate in your estimates about how many points your partners expect to receive from you?

- Nothing
- **You get a bonus added to your final payment**
- Your partners get a bonus added to their payments

## Experiment S5: Risk Game Instructions

Please read over the following instructions regarding the next decision-making task. If you have any questions, read back through these instructions. If you still have questions after reading through the instructions again, please get the experimenter before going any further.

In this task, you will be presented with different gambles and asked how much you would like to bet. You will complete this task by yourself and your decisions will not affect anyone else. Your results will also not be affected by anyone else.

For each round of this task, you will be given a number of points that you can use to place a bet. You will also be presented with a spinner wheel that has been divided into sections indicating how many points you would receive for every point you bet. For the gamble, the wheel will be spun and the section that the spinner stops on will determine how many points you receive. The sections of the wheel are labeled with "0 - 1 pts", "1 - 2 pts", "2 - 3 pts", and "3 - 4 pts". A random number will be selected from the range of the section that the spinner stops on and that amount will be multiplied by your bet to determine how much you receive. You may choose to bet some, all, or none of your initial point endowment. For example, if you bet 100 points and the spinner stops on "1 - 2 pts", a random number between 1 and 2 will be selected and your bet will be multiplied by that amount. In this case, if 1.5 is selected then you would receive 150 points. Your final points for each round will equal the number of points you did not bet plus the points you get from the gamble.

Treat the points in this task as you would real money because once the experiment has finished we will convert these points to real money and you will receive a payment corresponding to one of your decisions. The decision used to determine your payment will be randomly selected.

## Experiment S5: Risk Game Quiz

If you bet 100 points and the spinner landed on "2 - 3 pts", how many points would you receive?

- Zero points.
- **100 points multiplied by a random number between 2 and 3.**
- 100 points.

If you bet 50 points and the spinner landed on "0 - 1 pts", how many points would you receive?

- **An amount between 0 and 50 points.**
- An amount between 50 and 100 points.
- An amount between 100 and 150 points.

Will you have a partner for this task?

- Yes
- **No**
- Maybe

Results without Participant Exclusions

Experiment 2 (TG-I). Six participants answered questions at chance level and were excluded from analyses. Without excluding these 6 participants, there were no differences in the results. The drug X expectations interaction on investment was significant (*p* = .022) and the drug X expectations interaction on anticipated affective response was marginally significant (*p* = .066).

Experiment 3 (TG-I). Five participants answered questions at chance level and were excluded from analyses. Without excluding these 5 participants, there were no differences in the results. The drug X expectations interaction on investment was nonsignificant (*p* = .704) and the drug X expectations interaction on anticipated affective response was significant (*p* = .038).

Experiment 4 (TG-I with manipulated expectations). Five participants answered questions at chance level and were excluded from analyses. Without excluding these 5 participants, there were no differences in the results. The drug condition main effect on investment was significant (*p* = .047).

Experiment 5 (TG-I with manipulated expectations). Seven participants answered questions at chance level and were excluded from analyses. Without excluding these 7 participants, there were no differences in the results. The drug condition main effect on investment was nonsignificant (*p* = .335).

Experiment S3 (UG-R). Despite completing the quiz, five participants expressed confusion about the UG-R to the experimenter either during or after completion of the task and were, therefore, excluded from all UG-R analyses *a priori*. Furthermore, one participant was excluded for answering quiz questions at chance levels. When including this participant who performed below chance levels on the quizzes, the drug X change in proposal fairness interaction was marginal (*p* = .065). Without any exclusions, the interaction was a trend (*p* = .174).

Experiment S4 (TG-T). One participant was excluded from all TG-T and counterfactual guilt analyses for answering quiz questions at chance levels. Also, one participant was identified as having a high degree of influence on the TG-T model fit and excluded (Cook’s D = 0.13; calculated with the influence.ME package^7^). The drug X within-participant second-order beliefs interaction was marginal when including the participant with poor quiz performance (*p* = .09) and nonsignificant when including the one highly influential outlier (*p* = .23). The drug X within-participant counterfactual return interaction on guilt was still significant when including the participant for poor quiz performance (*p* = .04).

# References

1. Kahneman, D., Knetsch, J. L. & Thaler, R. H. Fairness and the Assumptions of Economics. *J. Bus.* **59**, S285–S300 (1986).

2. Güth, W., Schmittberger, R. & Schwarze, B. An experimental analysis of ultimatum bargaining. *J. Econ. Behav. Organ.* **3**, 367–388 (1982).

3. Durso, G. R. O., Luttrell, A. & Way, B. M. Over-the-counter relief from pains and pleasures alike: Acetaminophen blunts evaluation sensitivity to both negative and positive stimuli. *Psychol. Sci.* **26**, 750–758 (2015).

4. Crockett, M. J., Clark, L., Tabibnia, G., Lieberman, M. D. & Robbins, T. W. Serotonin modulates behavioral reactions to unfairness. *Science* **320**, 1739–1739 (2008).

5. Xiang, T., Lohrenz, T. & Montague, P. R. Computational substrates of norms and their violations during social exchange. *J. Neurosci.* **33**, 1099–1108 (2013).

6. Chang, L. J., Smith, A., Dufwenberg, M. & Sanfey, A. G. Triangulating the neural, psychological, and economic bases of guilt aversion. *Neuron* **70**, 560–572 (2011).

7. Nieuwenhuis, R., Pelzer, B. & Grotenhuis, M. te. *influence.ME: Tools for Detecting Influential Data in Mixed Effects Models*. (2015).

8. Singla, N. K. *et al.* Plasma and cerebrospinal fluid pharmacokinetic parameters after single‐dose administration of intravenous, oral, or rectal acetaminophen. *Pain Pract.* **12**, 523–532 (2012).
